# Genotypic Diversity and Pathogenic Potential of Clinical and Environmental *Vibrio parahaemolyticus* Isolates From Brazil

**DOI:** 10.3389/fmicb.2021.602653

**Published:** 2021-03-12

**Authors:** Leandro de O. Santos, Cristóvão A. de Lanna, Anna Carolina da C. Arcanjo, Paulo M. Bisch, Wanda M. A. von Krüger

**Affiliations:** Laboratório de Física Biológica, Instituto de Biofísica Carlos Chagas Filho, Universidade Federal do Rio de Janeiro, Rio de Janeiro, Brazil

**Keywords:** *Vibrio parahaemolyticus*, virulence markers, pandemic markers, infection models, murine, *Galleria mellonella*

## Abstract

*Vibrio parahaemolyticus* strains recovered from human diarrheal stools (one in 1975 and two in 2001) and environmental sources (four, between 2008 and 2010) were investigated for the presence of virulence genes (*trh*, *tdh*, and *vpadF*), pandemic markers (*orf8*, *toxRS*_new_), and with respect to their pathogenic potential in two systemic infection models. Based only on the presence or absence of these genetic markers, they were classified as follows: the environmental strains were non-pathogenic, whereas among the clinical strains, the one isolated in 1975 was pathogenic (non-pandemic), and the other two were pathogenic (pandemic). The pathogenic potential of the strains was evaluated in mice and *Galleria mellonella* larvae infection models, and except for the clinical (pathogenic, non-pandemic) isolate, the others produced lethal infection in both organisms, regardless of their source, serotype, and genotype (*tdh*, *orf8*, *toxRS*_new_, and *vpadF*). Based on mice and larval mortality rates, the strains were then grouped according to virulence (high, intermediate, and avirulent), and remarkably similar results were obtained by using these models: The clinical strain (pathogenic and non-pandemic) was classified as avirulent, and other strains (four non-pathogenic and two pandemic) were considered of high or intermediate virulence. In summary, these findings demonstrate that *G. mellonella* larvae can indeed be used as an alternative model to study the pathogenicity of *V. parahaemolyticus*. Moreover, they raise doubts about the use of traditional virulence markers to predict pathogenesis of the species and show that reliable models are indispensable to determine the pathogenic potential of environmental isolates considered non-pathogenic, based on the absence of the long-standing virulence indicators.

## Introduction

*Vibrio parahaemolyticus* is a Gram-negative halophilic bacterium, ubiquitous in estuarine and coastal waters, where it can be found free or in association with marine organisms, such as fish, shellfish, and zooplanktons (Baffone et al., 2006; Yu et al., 2013; Odeyemi, 2016; Lovell, 2017; Menezes et al., 2017; Narayanan et al., 2020; Parveen et al., 2020).

This species is recognized as a leading worldwide agent of acute gastroenteritis associated with the consumption of contaminated raw or undercooked seafood (Martinez-Urtaza et al., 2004; Leal et al., 2008; Letchumanan et al., 2014; Chen et al., 2017; Taylor et al., 2018). More rarely, it can cause wound infections that can lead to septicemia and death (Su and Liu, 2007; Tena et al., 2010; Huehn et al., 2014; Guillod et al., 2019).

Among the virulence factors in *V. parahaemolyticus*, the main ones are two hemolysins, the thermostable direct (TDH) (Nishibuchi and Kaper, 1995) and TDH-related (TRH) (Honda et al., 1988), encoded by the *tdh* and *trh* genes, respectively. Both hemolysins form pores in the target cell membrane, causing loss of ions and small molecules and an uncontrollable water influx (Yanagihara et al., 2010).

Other virulence-associated factors in *V. parahaemolyticus* are two type III secretion systems (T3SS1 and T3SS2) (Park et al., 2004; Hiyoshi et al., 2010) and two type VI secretion systems (T6SS1 and T6SS2) (Ho et al., 2014). These are protein complexes that form needle-like structures able to cross the host cell membrane to deliver bacterial effectors into the cytoplasm (Cornelis, 2006; Yu et al., 2012; Ceccarelli et al., 2013; Coulthurst, 2013). T3SS1 has been implicated in cell toxicity, and T3SS2 is involved in enterotoxicity, being required for the establishment of diarrhea in a model of orogastric infection in piglets (Park et al., 2004; Piñeyro et al., 2010; Ham and Orth, 2012). T6SS1, on the other hand, is an antibacterial system activated under warm marine conditions, whereas T6SS2 functions are unknown (Salomon et al., 2013).

Besides the factors mentioned earlier, there are pieces of evidence that many other proteins and toxins contribute to *V*. *parahaemolyticus* virulence (Cai and Ni, 1996; Krachler et al., 2011; Wang et al., 2013; Liu and Chen, 2015; Li et al., 2019). One of such proteins is the surface adhesion factor, VpadF, encoded by the *vp1767* gene, described as essential for lethality in intraperitoneally injected mice (Liu and Chen, 2015).

Although *tdh* and *trh* genes are the most used markers of *V. parahaemolyticus* virulence (Jones et al., 2012; Banerjee et al., 2014), not all clinical isolates are *tdh* and/or *trh* positive (Vongxay et al., 2008; García et al., 2009; Jones et al., 2012; Ceccarelli et al., 2013; Xu et al., 2015).

Moreover, these genes have also been detected in non-clinical isolates of the bacterium worldwide (Robert-Pillot et al., 2004; Johnson et al., 2012; Gutierrez West et al., 2013; Prabhakaran and Ramamurthy, 2020). Therefore, the pathogenesis of *V. parahaemolyticus* has not been fully elucidated, and other as yet unidentified virulence-related factors might be required for the establishment of infection.

The *V*. *parahaemolyticus* pathogenic strains have also been identified by their capsular (K) and lipopolysaccharide (O) antigens or serotype (Nair et al., 2007; Alam et al., 2009; Broberg et al., 2011; Le Roux et al., 2015; Raszl et al., 2016). Until 1996, *V. parahaemolyticus* cases of infection were sporadic, occurred in certain countries, and could be related to diverse serovars (Bhuiyan et al., 2002). However, at the beginning of 1996, an atypical outbreak of *V. parahaemolyticus* infection occurred in India, and it was linked to strains of the new serotype O3:K6, which carry the specific genetic markers *tdh*, *toxRS*_new_ (Matsumoto et al., 2000), and *orf8* (Nasu et al., 2000). Subsequently, isolates similar to those found in India, belonging to O3:K6 serotype or related serovars (Matsumoto et al., 2000), have been identified in outbreaks of human gastroenteritis in many countries around the five continents (Velazquez-Roman et al., 2014), indicating the pandemic character of these strains.

The first cases of *V*. *parahaemolyticus* infection in South America occurred in Brazil in 1975, affecting adults and children. The agent was isolated, serotyped as O5:K17 and denominated Cascavel (Hofer, 1983), and was one of the clinical strains analyzed in this work. In the following years, several *V. parahaemolyticus* outbreaks of gastroenteritis have been reported in the country (Magalhães et al., 1991; Leal et al., 2008), as well as cases of isolation of the bacterium from bivalve mollusks for human consumption and seawater from oyster farming areas (Rojas et al., 2011; Sobrinho et al., 2011; Ramos et al., 2012; Menezes et al., 2017).

Moreover, many studies have provided evidence on the increase and spread of *Vibrio* species worldwide due to sea surface warming over the last decades and on an unprecedented frequency and severity of human infections caused by these organisms (Martinez-Urtaza et al., 2010; Raszl et al., 2016; Vezzulli et al., 2016; Baker-Austin et al., 2017; Galanis et al., 2020). These factors pose a risk of further outbreaks of gastroenteritis or other infections by *V. parahaemolyticus* all over the world.

Despite the increasing number of reports on *V. parahaemolyticus* in seafood and clinical specimens in Brazil, few studies have investigated the relationship between the pathogenic potential and genotype of the strains isolated in the country.

Therefore, in the present work, we analyzed the genotypic diversity of *V. parahaemolyticus* strains isolated from clinical and environmental sources in Brazil, focusing on virulence and pandemic marker genes and their pathogenic potential using two models of systemic infection.

## Materials and Methods

### Bacterial Isolates, Culture Media, and Conditions

Seven *V. parahaemolyticus* strains isolated in Brazil were analyzed in the present work: Four environmental, obtained from samples collected in an oyster farming area on the south coast of the country (Palhoça, Santa Catarina), and three clinical, isolated from fecal samples of patients with diarrhea clinical in the northeast of Brazil (Ceará and Pernambuco). It is worth mentioning that one of the clinical isolates analyzed in this work, Cascavel, was involved in the first cases of *V. parahaemolyticus* gastroenteritis in Latin America (Brazil, in 1975). Detailed information about the strains is listed in Table 1.

**TABLE 1:** *Vibrio parahaemolyticus* strains isolated in Brazil and analyzed in this study.

Strains	Source Local of isolation^*b*^	Year of isolation	Serotype (O:K)^*a*^	References
Cascavel	Clinical	1975	O5:K17	Hofer, 1983
	Cascavel, CE			
IOC 17381	Clinical	2001	O3:KUT	FIOCRUZ-RJ
	Recife, PE			
IOC 17384	Clinical	2001	O3:KUT	Leal et al., 2008
	Recife, PE			
IOC 20173	Environmental (seawater)	2010	O1:K25	FIOCRUZ-RJ
	Palhoça, SC			
IOC 20142	Environmental (seawater)	2008–2009	O1:KUT	Ramos et al., 2012
	Palhoça, SC			
IOC 20128	Environmental (oyster)	2010	OUT:KUT	FIOCRUZ-RJ
	Palhoça, SC			
IOC 20138	Environmental (seawater)	2008–2009	OUT:KUT	Ramos et al., 2012
	Palhoça, SC			

*^*a*^“UT” stands for untypeable.*

*^*b*^Environmental strains isolated in an oyster farm: three from seawater samples and one from an oyster.*

All *V. parahaemolyticus* isolates (Table 1) were provided by Dr. Dália P. Rodrigues, from Fundação Oswaldo Cruz (FIOCRUZ-RJ), Rio de Janeiro, Brazil.

For the infection assays, spontaneous streptomycin-resistant (SR) mutants were used. They were selected by plating overnight cultured cells on a solid medium (below) containing streptomycin at 100 μg/ml. One SR colony from each strain was randomly selected and named: CascavelSR, IOC 17381SR, IOC 17384SR, IOC 20173SR, IOC 20142SR, IOC 20128SR, and IOC 20138SR.

*Vibrio parahaemolyticus* reference strains RIMD2210633 (GenBank accession numbers BA000031 and BA000032) (Makino et al., 2003), a pandemic isolate, serotype O3:K6 and BB22OP (GenBank accession numbers CP003972 and CP003973) (Jensen et al., 2013), a pre-pandemic environmental isolate from early 1980, O4:K8 serotype, were used as sources of operon *toxRS*_new/old_ and gene *vpadF* sequences. The strains A431 (*tdh*^+^) and 0798081 (*trh*^+^) were isolated in Brazil, respectively, from a clinical specimen and a fish (Fundação Oswaldo Cruz, Rio de Janeiro) and were used as positive controls for *tdh* and *trh* genes in polymerase chain reaction (PCR) assays (item 2.3).

Bacteria were routinely cultured at 37°C, in Lysogeny broth (LB) liquid medium (Bertani, 1951) at 200 rpm or on LB-agar (1.5%). Sodium chloride to a final concentration of 2.2% (FDA, 2004; Nishina et al., 2004) and streptomycin at 100 μg/ml were also added to both media (Sambrook et al., 1989).

### Determination of Hemolytic Activity: Kanagawa Phenomenon

The seven *V. parahaemolyticus* strains were grown in LB to OD_600*nm*_ 0.5–0.6, and the Kanagawa phenomenon was assayed on Wagatsuma blood agar (Wagatsuma, 1968). Briefly, 10 μl of each of the seven cultures was spotted on modified blood agar, prepared as previously described (Okuda and Nishibuchi, 1998), and incubated overnight at 37°C. The Kanagawa phenomenon was considered positive when a halo due to β-hemolysis was observed around the bacterial growth. For statistically significant results, each culture was spotted on the Wagatsuma blood agar at least 10 times.

### Genomic DNA Preparation, PCR Assays, and Sequencing

Genomic DNA from the *V. parahaemolyticus* strains was prepared from cells grown in LB liquid medium for 14–16 h. The cells were collected by centrifugation at 12,000 × *g* and 4°C for 5 min, and the DNA samples were extracted using the Wizard Genomic DNA Purification^®^ kit (Promega), according to the manufacturer’s instructions. These DNAs were used as templates for the PCRs described later.

Polymerase chain reactions were used to investigate the presence of *tlh*, *tdh*, and *trh* genes in the genome of all strains with gene-specific primer pairs (Table 2) and the conditions proposed by the United States Food and Drugs Administration, as described previously (Bej et al., 1999).

**TABLE 2:** Oligonucleotide primers used in this study.

Gene	Amplified fragment (bp)	Oligonucleotides	Sequences	References
*tlh*	450	L-TL	**5′** AAA GCG GAT TAT GCA GAA GCA CTG **3′**	Bej et al., 1999
		R-TL	**5′** GCT ACT TTC TAG CAT TTT CTC TGC **3′**	
*tdh*	270	VPTDH-L	**5′** GTA AAG GTC TCT GAC TTT TGG AC **3′**	Bej et al., 1999
		VPTDH-R	**5′** TGG AAT AGA ACC TTC ATC TTC ACC **3′**	
*trh*	500	VPTRH-L	**5′** TTG GCT TCG ATA TTT TCA GTA TCT **3′**	Bej et al., 1999
		VPTRH-R	**5′** CAT AAC AAA CAT ATG CCC ATT TCC G **3′**	
*orf8*	746	Orf8 Fw	**5′** GTT CGC ATA CAG TTG AGG **3′**	Iida et al., 2001
		Orf8 Rv	**5′** AAG TAC AGC AGG AGT GAG **3′**	
*toxRS*	1470	toxRS.1	**5’** TAT CTC CCA TGC GCA AAC GTA **3’**	Lin et al., 1993
		toxRS.2	**5’** ACA GTA CCG TAG AAC CGT GAT **3’**	
*vpadF*	2,560	VpadF-F	**5′** GCG AAT TGA GCA CTT CCC ATT AC **3′**	Liu and Chen, 2015
		VpadF-R	**5′** CCT TAC TTA AGA GGA ACG CCA G **3′**	

*bp, base pairs.*

For *orf8* detection (746-bp fragment), primers (Table 2) and PCR conditions used were those described previously (Iida et al., 2001).

The *toxRS* operon from each isolate was amplified by PCR (primers in Table 2) and sequenced as previously described (Matsumoto et al., 2000). Briefly, the PCR products obtained using primers were analyzed by electrophoresis in 1% agarose gel in Tris acetate-ethylenediaminetetraacetic acid 1 × (Sambrook et al., 1989), and the 1,470-bp fragments were purified with the Illustra GFX PCR DNA and gel band purification kit (GE Healthcare), according to the manufacturer’s instructions. Approximately 25 ng of each fragment was used as a template with the PCR primers to determine the sequence in both directions, by the Sanger method, using the ABI Prism 3100 Genetic Analyzer (Applied Biosystems). The *toxRS* operon sequences of the seven strains were aligned with those reference strains RIMD2210633 (Makino et al., 2003) and BB22OP (Jensen et al., 2013), that carry, respectively, operon *toxRS*_new_ and *toxRS*_old_ (GenBank accession numbers: BA000031 and CP003972, respectively) (Lin et al., 1993). These sequences were obtained from the National Center for Biotechnology Information (NCBI) database and compared with the CLUSTAL Omega sequence alignment tool (Larkin et al., 2007; Sievers et al., 2011).

DNA fragments (2,560 bp) containing the *vp1767* gene were PCR amplified from the genome of the seven strains, using primers VpadF-F and VpadF-R (Table 2) as previously described (Liu and Chen, 2015). They were purified and sequenced, and *vp1767* sequences were then compared among themselves and with those from the *V. parahaemolyticus* reference strains RIMD2210633 and BB22OP, as described earlier.

### Murine Infection Model

Male Swiss mice aged 5–6 weeks and weighing 30–50 g were used to evaluate clinical signs and mortality, as previously described (Hiyoshi et al., 2010). Briefly, bacterial cells were grown to the exponential phase, and an aliquot of 100 μl containing 10^8^ or 10^6^ CFU of each of the seven strains was inoculated intraperitoneally per mouse, using disposable 1-ml syringes with 13 × 0.45-mm needles. Ten animals were used per dose of each strain, and control mice were inoculated with 100 μl of pure, sterile LB (*n* = 5 animals).

At 24-, 48-, 72-, 96-, and 120-h post-inoculations (p.i.), 11 clinical signs of systemic infection were assessed using SHIRPA primary screen, as well as mortality. The clinical signs considered were piloerection, contracted abdomen, stool changes, lacrimation, eyelid closure, alteration of locomotor activity, body temperature change, interest in the environment, grabbing force, change in respiration rate, and alert (escape to the touch) (Rogers et al., 2001; Lackner et al., 2006).

For each sign, one point was given, and the sum of these points or clinical scores per mouse was calculated. To obtain the mean clinical score value every 24-h p.i., the sum of scores of all mice in a group was divided by the number of animals in that group. The mean clinical score reflected the state of health of animals, as follows: healthy mice, score zero; mice with a mild infection, scores lower than 3; mice with moderate infection, scores from 3 to 7; and mice with severe infection, scores 8–11 (Rogers et al., 2001). These score values were submitted to the two-way analysis of variance test (Yates, 1934). To evaluate the evolution of the clinical status of the mice in each group over the 120-h p.i., the Tukey (1949) test was used, whereas the comparison between each group and the control group was made by the Dunnett (1955) test. For all tests, *p*-values < 0.05 were considered statistically significant.

In addition to evaluating clinical signs, survival curves of animals in each group were generated using the Kaplan–Meier method (Kaplan and Meier, 1958) and compared using the log-rank test (Mantel, 1966; Peto and Peto, 1972), with *p*-values < 0.05 considered statistically significant.

All experiments were done according to protocol number 010/16, approved by the Comissão de Ética no Uso de Animais of the Centro de Ciências da Saúde of the Universidade Federal do Rio de Janeiro (UFRJ).

### *Galleria mellonella* Infection Model

The pathogenic potential of the seven *V. parahaemolyticus* isolates was also evaluated in *G. mellonella* moth larvae through intra-hemocelic inoculation of bacterial suspension, as previously described (Ramarao et al., 2012).

Briefly, the sixth and seventh instars (weighing from 200 to 250 mg each) were cleaned with 70% ethanol and transferred to a microtube on ice for 5 min to anesthetize. Each larva was then inoculated with *V. parahaemolyticus* cells in the exponential phase of growth, through the last left proleg using a Hamilton syringe with a 31-gauge 8-mm long needle.

The inoculum dose ranged from 10^3^ to 10^6^ CFU/10 μl per larva, and a group of 20 larvae was used per dose of each of the seven *V. parahaemolyticus* strains. Three control groups (*n* = 10 per group) were used: uninoculated larvae, larvae inoculated with 10 μl of sterile phosphate-buffered saline (PBS), and larvae inoculated with 10^8^ CFU/10 μl of each strain, killed by heating at 100°C for 30 min (heat-killed bacteria). Inoculated larvae were maintained at 37°C for 120 h, under protection from light, and mortality was evaluated every 24-h p.i.

To differentiate dead from living larvae, their color (living larvae are whitish, and the dead ones are dark brown) and ability to move and to respond to physical stimuli were observed, as previously suggested (Ramarao et al., 2012). Survival curves of larvae in each group were generated using the Kaplan–Meier method (Kaplan and Meier, 1958) and compared using the log-rank test (Mantel, 1966; Peto and Peto, 1972), with *p*-values < 0.05 considered statistically significant.

## Results

### Virulence and Pandemic Marker Genes in *Vibrio parahaemolyticus* Strains

The strains (Cascavel, IOC 17381, IOC 17384, IOC 20173, IOC 20142, IOC20128, and IOC 20138) (Table 1) were investigated for the presence of the *tlh* gene, for the thermolabile hemolysin (TLH) that has been used as a signature molecular marker for *V. parahaemolyticus* and for the virulence-related genes *tdh* and *trh*, coding, respectively, for the TDH and the TRH hemolysins. The *tlh* gene was found in all strains, based on the PCR amplification of a 450-bp fragment containing the gene, confirming they all are true of the species (Supplementary Figure 1A). Interestingly, only the clinical isolates (Cascavel, IOC 17381, and IOC 17384) showed PCR amplification for the *tdh* gene fragment (270 bp) (Supplementary Figure 1B) and were positive for β-hemolysis on Wagatsuma agar (data not shown), known as the Kanagawa phenomenon (KP), that has been related to the hemolytic activity in *V. parahaemolyticus* TDH-positive strains (Zen-Yoji et al., 1971; Niikawa et al., 1972). None of the seven strains was positive for *trh* gene (Supplementary Figure 1C).

The genomes of the seven strains were also investigated for the presence of the open reading frame 8 (*orf8*) and *toxRS* operon, as both have been specifically associated with pandemic isolates of *V. parahaemolyticus*.

Gene *orf8* that encodes a putative cell surface adhesion protein is a unique open reading frame, only found in the filamentous phage f237 genome, which has been exclusively associated with recent *V. parahaemolyticus* O3:K6 strains (Nasu et al., 2000). Interestingly, among the seven strains, only the *tdh*-positive clinical strains, Cascavel (O5:K17), IOC 17381, and IOC 17384 (both O3:KUT), carried gene *orf8* (data not shown).

The *toxRS* operon, on the other hand, is well conserved in the *Vibrio* genus and encodes the global regulator ToxR that directly activates transcription of major virulence factors genes in some species, including *V. parahaemolyticus* (Lin et al., 1993). The new *V. parahaemolyticus* O3:K6 strains carried a *toxRS* operon sequence (*toxRS*_new_) that differs from that of the old O3:K6 strains (*toxRS*_old_), isolated before 1995, in seven base positions (Matsumoto et al., 2000). The presence of *toxRS*_new_ or *toxRS*_old_ in the genomes of the seven *V. parahaemolyticus* strains was then investigated by PCR and sequencing, as previously described (Matsumoto et al., 2000). The *toxRS*_new_ sequence was only found in the clinical strains IOC 17381 and IOC 17384, isolated in 2001 (Table 3). The clinical strain Cascavel isolated in 1975, well before the emergence of the pandemic clone in 1996 (Okuda et al., 1997), carried a copy of the *toxRS*_old_ operon (Table 3) as the non-pandemic isolates.

**TABLE 3:** Bases in seven positions within the 1,589-bp *toxRS* sequence of *Vibrio parahaemolyticus* strains.

Strains	Base position in *toxRS* sequence (1,589 bp)^*a*^							*toxRS* type^*b*^	Serotype (O:K)^*c*^
	
	576	900	1,002	1,196	1,214	1,244	1,463		
Cascavel	G	G	C	C	A	**A**	A	old	O5:K17
IOC 17381	**A**	**A**	**T**	**T**	**T**	**A**	**T**	**new**	O3:KUT
IOC 17384	**A**	**A**	**T**	**T**	**T**	**A**	**T**	**new**	O3:KUT
IOC 20173	G	G	C	C	A	G	A	old	O1:K25
IOC 20142	G	G	C	C	A	G	A	old	O1:KUT
IOC 20128	G	G	**T**	**T**	**T**	G	A	hybrid	OUT:KUT
IOC 20138	G	G	C	C	A	G	A	old	OUT:KUT
RIMD2210633	**A**	**A**	**T**	**T**	**T**	**A**	**T**	**new**	O3:K6
BB22OP	G	G	**T**	C	A	G	A	old	O4:K8

**Vibrio parahaemolyticus* reference strains for *toxRS*_new_ and *toxRS*_old_, respectively: pandemic RIMD2210633 and non-pandemic BB22OP.*

*Bases in the *toxRS*_new_ sequence that differ from those in *toxRS*_old_ are in bold.*

*^*a*^The seven bases in the *toxRS*_new_ sequence that differ from those in *toxRS*_old_ are in bold.*

*They were numbered (576, 900, 1,002, 1,196, 1,214, 1,244, and 1,463) based on *toxRS* sequence of *V. parahaemolyticus* strain AQ3815 (Lin et al., 1993).*

*^*b*^The *toxRS* types “old,” “new,” or “hybrid” are based on Matsumoto et al. (2000).*

*^*c*^“UT” stands for untypeable.*

Among the four environmental strains (*tdh*^–^ and *orf8*^–^), three of them, IOC 20173, IOC 20142, and IOC 20138, isolated between 2008 and 2010 from seawater samples, carried one copy of *toxRS*_old_ each (Table 3). Surprisingly, in the strain IOC 20128, isolated in 2010 from a farmed oyster, the *toxRS* operon sequence was a hybrid between *toxRS*_old_ and *toxRS*_new_ (Matsumoto et al., 2000). Four of the seven divergent bases matched those in *toxRS*_old_ sequence (576, 900, 1,244, and 1,463 positions), and the other three (1,002, 1,196, and 1,214 positions) were identical to those in *toxRS*_new_ sequence (Table 3). As far as we know, this is the first report of a hybrid *toxRS* operon sequence in *V. parahaemolyticus*.

The surface protein VpadF has been described as a major virulence factor of *V. parahaemolyticus*, essential for lethality in intraperitoneally injected mice (Liu and Chen, 2015). However, PCR and sequencing showed that the seven isolates carry the *vpadF* gene (*vp1767*). Alignment of the vpadF gene sequences with those of the reference strains RIMD2210633 and BB22OP (Supplementary Figure 2) showed genes equal in length (2,211 bp), and the predicted VpadF protein had 736 amino acids for all strains. Moreover, a pairwise comparison of *vpadF* sequences and VpadF deduced amino acid sequences from the nine strains showed, in both cases, high identity ranging from 98 to 100% (Supplementary Figure 2 and Supplementary Table 1).

### Pathogenic Potential of *Vibrio parahaemolyticus* Strains: Murine Infection Assay

Although *V. parahaemolyticus* is better known for causing gastroenteritis, it can also cause wound infections that can advance to septicemia and death (Daniels et al., 2000; Su and Liu, 2007; Tena et al., 2010; Huehn et al., 2014; Guillod et al., 2019).

Therefore, we decided to investigate the pathogenic potential of the seven *V. parahaemolyticus* strains in the well-established murine model of systemic infection (Hiyoshi et al., 2010).

Mortality and clinical condition of mice injected intraperitoneally with distinct doses (number of CFUs injected/mouse) of each of the seven *V. parahaemolyticus* strains were monitored every 24 h for 120 h (post-infection, p.i.). Eleven simple tests were carried out to obtain behavioral and functional profiles of infected mice and control animals (Rogers et al., 2001).

**FIGURE 1:**
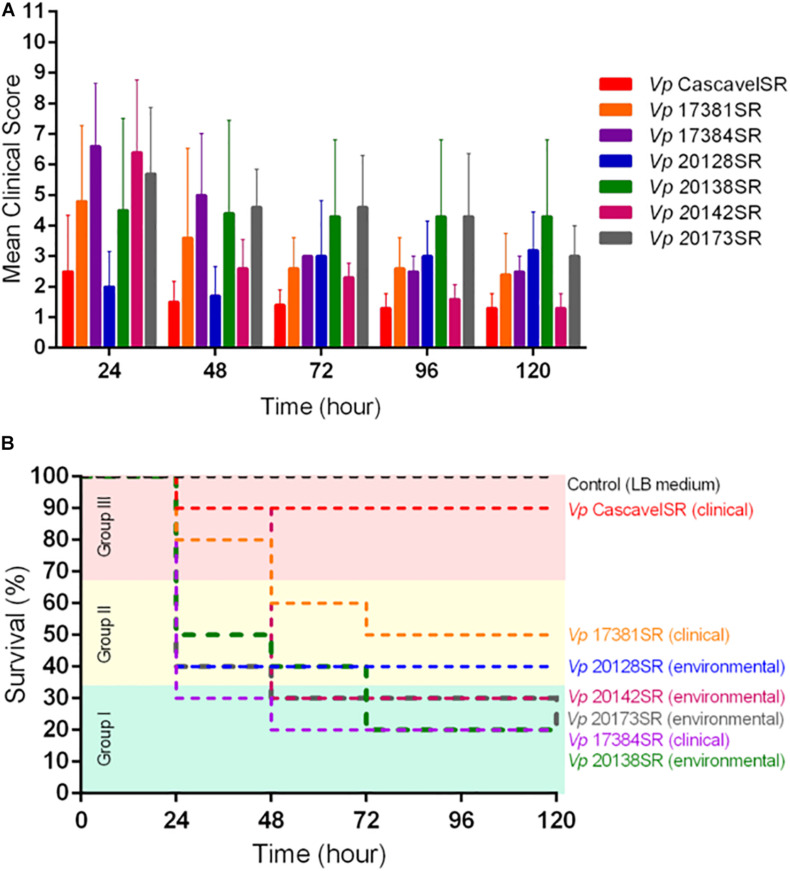
Mean clinical scores **(A)** and survival curves **(B)** of mice inoculated with each of the seven *Vibrio parahaemolyticus* strains at a dose of 10^8^ CFU/animal. Eleven clinical signs were evaluated every 24 h for 120 h post-infection, in groups of *n* = 10 animals per dose of each strain. Control group: mice inoculated with LB medium, *n* = 5 animals. Strains were grouped according to virulence: group I (green) – high; group II (yellow) – intermediate; and group III (red) – low. Survival curves of mice in each group were generated using the Kaplan–Meier method (Kaplan and Meier, 1958) and compared using the log-rank test (Mantel, 1966; Peto and Peto, 1972), with *p*-values < 0.05 considered statistically significant.

Based on the mean clinical scores, we concluded that the severity of the disease was dose-dependent, regardless of the bacterial strain tested. Among mice inoculated with 10^8^ CFU/animal, for most of the seven strains, the highest mean clinical scores were observed 24-h p.i. (Figure 1A). Moreover, the higher these scores, the higher were mice mortality rates during the experimental timeline, which for most of the strains varied between 50 and 80% and were independent of the strain source, serotype, and presence of *tdh*, *orf8*, and *toxRS*_new_ genes (Figures 1A,B). The only exception was the clinical strain Cascavel (O5:K17, *tdh*^+^, and *orf8*^+^), which at this inoculum dose caused few signs of illness (Figure 1A) and very low mortality rate (10%) in mice, not statistically significant in comparison with the control (zero death) (*p* < 0.05) (Figure 1B).

Inoculum dose of 10^6^ CFU/animal of all the seven strains, on the other hand, resulted in an insignificant mice mortality (10% in 120 h; *p* < 0.05), comparable with the control group (Supplementary Figure 3).

Taken together, the results mentioned earlier showed a positive correlation between the severity of clinical condition 24-h p.i. and the mortality rates of mice inoculated with 10^8^ CFU/animal of each strain, corroborating data of previous studies on animal sepsis induced by other bacterial species (Shrum et al., 2014; Gonçalves et al., 2017).

### Pathogenic Potential of *Vibrio parahaemolyticus* Strains: *Galleria mellonella* Infection Assay

We also tested larvae of *G. mellonella* as an alternative model to study systemic infection by the seven *V. parahaemolyticus* strains.

To this end, larvae were challenged with different doses (10^3–6^ CFUs/larva) of each strain, and mortality was followed every 24 h for 120 h. For all strains, independently of the origin of isolation and genotype, we observed dose-dependent larval mortality.

Doses of 10^5^ and 10^6^ CFUs/larva of the clinical IOC 17381SR, IOC 17384SR, and environmental isolates IOC 20173SR, IOC 20142SR, IOC 20128SR, and IOC 20138SR led to high larval mortality in 120 h (65–100%, *p* < 0.05). The clinical strain CascavelSR (O5:K17, *tdh*^+^, and *orf8*^+^), on the other hand, at such doses killed, respectively, 20 and 50% of the inoculated larvae (Supplementary Figure 4).

**FIGURE 2:**
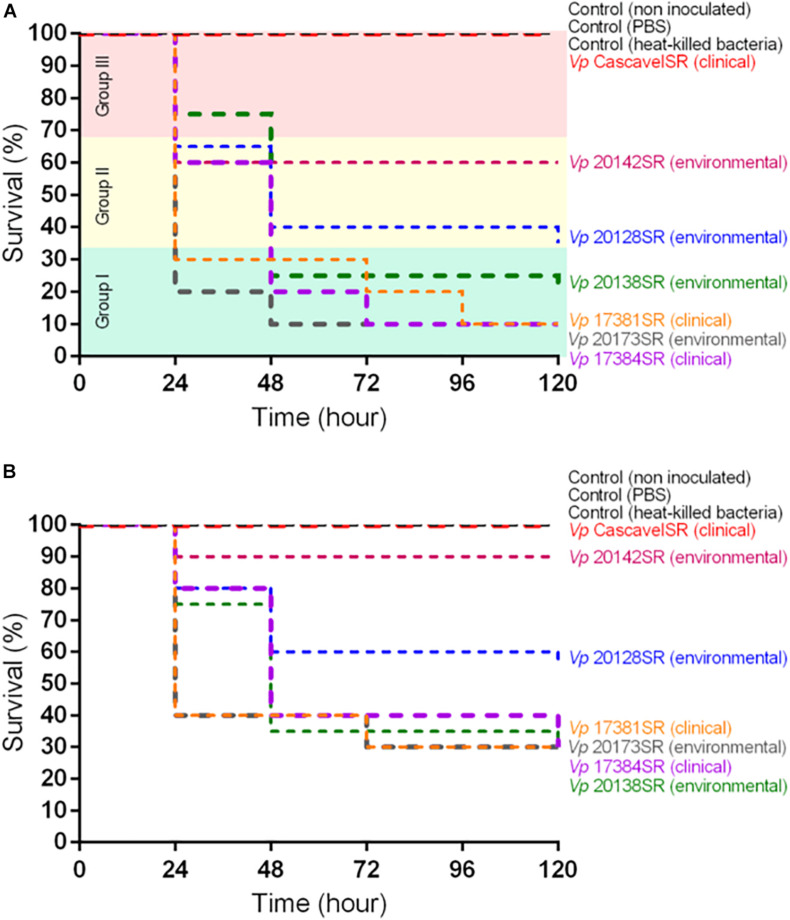
Survival curves of *Galleria mellonella* larvae inoculated with **(A)** 10^4^ CFU/larva and **(B)** 10^3^ CFU/larva of each of the seven *Vibrio parahaemolyticus* strains. Each group contained *n* = 20 larvae per dose of each strain. Three controls, with *n* = 10 larvae per group, were used: non-inoculated, inoculated with phosphate-buffered saline, and heat-killed bacteria. Strains were grouped according to virulence: group I (green) – high; group II (yellow) – intermediate; and group III (red) – low. Survival curves of larvae in each group were generated using the Kaplan–Meier method (Kaplan and Meier, 1958) and compared using the log-rank test (Mantel, 1966; Peto and Peto, 1972), with *p*-values < 0.05 considered statistically significant.

However, inoculation with 10^4^ CFU/larva allowed us to detect important strain-dependent differences in the larval mortality rate (Figure 2A).

Inoculation with 10^3^ CFU/larva leads to lower larval mortality rates (10–70%) but confirmed the grouping of the strains based on the inoculum dose of 10^4^ CFU/larva, except the environmental strain IOC 20142SR that grouped with CascavelSR (Figure 2B).

## Discussion

The first cases of *V*. *parahaemolyticus* gastroenteritis in Brazil occurred in 1975 and were caused by strain Cascavel, serotyped as O5:K17 (Hofer, 1983), which we had the opportunity to analyze in this study. Since 1975, there have been many reports on *V. parahaemolyticus* gastroenteritis outbreaks (Magalhães et al., 1991; Leal et al., 2008) and on the isolation of the bacterium from environmental sources in the country (Rojas et al., 2011; Ramos et al., 2012; Menezes et al., 2017). However, few studies have investigated the relationship between the pathogenic potential and genotype of these strains.

Therefore, in the present work, we analyzed the genotypic diversity of seven *V. parahaemolyticus* isolates from clinical and environmental sources in Brazil (Table 1), focusing on virulence and pandemic marker genes (Table 4) and also on their pathogenic potential using two models of systemic infection, the murine and larvae of *G*. *mellonella* (Table 5).

**TABLE 4:** Classification of the *Vibrio parahaemolyticus* strains analyzed in this work based on phenotypic and genotypic characteristics.

Strains	Source	Serotype (O:K)^*b*^	*tdh*	*trh*	*orf8* (*f237*)	*toxRS*_new_	Classification^*a*^
Cascavel	Clinical (fecal sample)	O5:K17	+	–	+	–	Pathogenic (non-pandemic)
IOC 17381	Clinical (fecal sample)	O3:KUT	+	–	+	+	Pathogenic (pandemic)
IOC 17384	Clinical (fecal sample)	O3:KUT	+	–	+	+	Pathogenic (pandemic)
IOC 20173	Environmental (seawater)	O1:K25	–	–	–	–	Non-pathogenic
IOC 20142	Environmental (seawater)	O1:KUT	–	–	–	–	Non-pathogenic
IOC 20128	Environmental (oyster)	OUT:KUT	–	–	–	Hybrid	Non-pathogenic
IOC 20138	Environmental (seawater)	OUT:KUT	–	–	–	–	Non-pathogenic

*^*a*^Classification as proposed by Velazquez-Roman et al. (2012).*

*^*b*^“UT” stands for untypeable.*

*+, present; –, absent; and hybrid, four of the seven divergent bases match those in *toxRS*_old_, and three match those in *toxRS*_new_.*

**TABLE 5:** Classification of the *Vibrio parahaemolyticus* strains analyzed in this work based on phenotypic and genotypic characteristics and virulence in mice and *Galleria mellonella* models of infection.

Strains	Source	Classification	
		
		(Virulence and pandemic genes)^*a*^	(virulence in mice/larvae)
Cascavel	Clinical	Pathogenic (non-pandemic)	Avirulent
IOC 17381	Clinical	Pathogenic (pandemic)	Intermediate/high
IOC 17384	Clinical	Pathogenic (pandemic)	High
IOC 20173	Environmental	Non-pathogenic	High
IOC 20142	Environmental	Non-pathogenic	High/intermediate
IOC 20128	Environmental	Non-pathogenic	Intermediate
IOC 20138	Environmental	Non-pathogenic	High

*^*a*^Classification as proposed by Velazquez-Roman et al. (2012).*

As expected, only the three clinical *V. parahaemolyticus* isolates were *tdh* positive (Table 4), a finding that correlates well with the observations that the *tlh/tdh* combination is more prevalent than *tlh/trh* among *V. parahaemolyticus* isolates (Bej et al., 1999), that *tdh* is more commonly found in clinical than in environmental isolates of *V. parahaemolyticus* (Okuda et al., 1997; Ceccarelli et al., 2013) and that the hemolysin TDH is a major factor for the gastrointestinal illness caused by this species (Hiyoshi et al., 2010; Letchumanan et al., 2014).

Curiously, the *orf8* gene, which has been considered exclusive of the recent *V. parahaemolyticus* O3:K6 strains (Nasu et al., 2000), has been found in the clinical strain Cascavel, isolated in 1975, well before the emergence of the pandemic clone in 1996 (Okuda et al., 1997). The *toxRS*_new_ operon, another important feature of the pandemic clones (Matsumoto et al., 2000; Nair et al., 2007), was detected only in the clinical strains IOC 17381 and IOC 17384 (O3:KUT, *tdh*^+^, and *orf8*^+^) isolated in 2001. Strain Cascavel (O5:K17, *tdh*^+^, and *orf8*^+^), isolated in 1975, on the other hand, had a copy of the *toxRS*_old_ operon, as the non-pandemic strains (Lin et al., 1993; Matsumoto et al., 2000). The fact that Cascavel shared phenotypic (O5:K17, a serovariant of O3:K6) and genetic (*tdh* and *orf8* genes) characteristics with pandemic isolates is intriguing and corroborates the hypothesis that neither *orf8* gene nor serovar can be considered reliable pandemic group-specific marker, as previously observed (Bhuiyan et al., 2002; Okura et al., 2003; Chowdhury et al., 2004; Meador et al., 2007).

The environmental isolates, IOC 20173 (O1:K25), IOC 20142 (O1:KUT), and IOC 20138 (OUT:KUT), also belonged to serotypes regarded as serovariants of pandemic O3:K6 (Nair et al., 2007) but were negative for the virulence related-genes *trh* and *tdh* and pandemic markers *orf8* and *toxRS*_new_ (Table 4), casting further doubt on the value of serotype as a reliable indicator of a pandemic isolate.

Based on the presence or absence of virulence genes (*tdh* and *trh*) and pandemic marker genes (*orf8*, *toxRS*_new_), the seven *V. parahaemolyticus* isolates were then classified, as previously proposed (Velazquez-Roman et al., 2012). The four environmental strains (*tdh*^–^, *orf8*^–^, and *toxRS_new_^–^*) were non-pathogenic, whereas, among the clinical strains, Cascavel (O5:K17, *tdh*^+^, and *orf8^+^)* was pathogenic (non-pandemic), and IOC 17381 and IOC 17384 (O3:KUT, *tdh*^+^, *orf8*^+^, and *toxRS*_new_^+^) were pathogenic (pandemic) isolates (Table 4).

Analysis of the pathogenic potential of the strains in intraperitoneally injected mice and *G. mellonella* larvae showed that, with exception to the pathogenic (non-pandemic) strain Cascavel, the other six strains caused infection and death, regardless of their origin, serotype, and presence of *tdh*, *orf8*, *toxRS*_new_, and *vpadF* genes.

Moreover, based on the mortality rates of mice inoculated with 10^8^ CFU/animal (Figure 1B), the strains were grouped in decreasing order of virulence, as follows. Group I (high virulence) included the clinical IOC 17384 and the environmental IOC 20173, IOC 20142, and IOC 20138 that killed ≥70% of mice in 120-h p.i. (*p* > 0.05). Group II (intermediate virulence) included the environmental strain IOC 20128 and the clinical IOC 17381 that caused mice mortality of, respectively, 60 and 50% 120-h p.i. (*p* < 0.05), and group III (avirulent) was the clinical strain Cascavel that caused insignificant mice mortality when compared with the control (Table 5).

A remarkably similar grouping was obtained based on mortality rate data of *G. mellonella* larvae inoculated with 10^4^ CFU/larva of each of the seven strains (Figure 2A). Group I (high virulence) included the clinical strains IOC 17381, IOC 17384, and the environmental isolates IOC 20173 and IOC 20138 that killed ≥70% of the larvae in 120-h p.i. (*p* > 0.05). Group II (intermediate virulence) was formed by the environmental strains IOC 20142 and IOC 20128, which caused larval mortality of, respectively, 40 and 65% in 120-h p.i. (*p* < 0.05), and group III (avirulent) included strain Cascavel that, differently from the other isolates (*p* < 0.05), did not kill the inoculated larvae in the timeline of the experiment (Table 5).

The only difference between the two groupings was the assignment of the clinical IOC 17381 and of the environmental IOC 20142 strains to groups (Table 5), which is likely due to a strain-specific response to each host environment.

Thus, except for clinical strain Cascavel, classified as avirulent to mice and *G. mellonella* larvae, the other six strains (two clinical and four environmental) were considered of high or intermediate virulence in these models. It is worth noting that among the four most virulent strains (group I, in mice/larvae), three/two were isolated from environmental sources, whereas among the three clinical strains, one was considered avirulent (group III) and the other two of high/intermediate virulence (groups I/II) (Table 5).

The fact that the four environmental strains (*tdh*^–^, Table 4) showed virulence in mice and larvae, whereas the clinical Cascavel (*tdh*^+^, Table 4) did not, strongly indicates that TDH is not essential for the bacterium to establish systemic infection, corroborating previous findings (Hoashi et al., 1990; Mahoney et al., 2010; Jones et al., 2012; Wagley et al., 2018), and highlights the pathogenic potential of the environmental strains analyzed in this study.

It is important to emphasize that this work showed for the first time a very good correlation between the virulence of *V. parahaemolyticus* strains measured in mice and *G. mellonella*, indicating that this insect larva can be successfully used as an alternative and inexpensive model to study the pathogenicity of *Vibrio* species, as reported (Nuidate et al., 2016; Bokhari et al., 2017; Wagley et al., 2018).

Another particularly important finding of this study was that the two clinical isolates IOC 17381 and IOC 17384 (pathogenic/pandemic and virulent), in contrast to the clinical strain Cascavel (pathogenic/non-pandemic and avirulent), can cause both systemic infection and gastroenteritis. As these three strains were isolated in the same region of the country, but approximately 35 years apart (Table 1), a comparative genomic analysis would provide a better understanding of their distinct pathogenic potential, environmental adaptation, and evolution.

In conclusion, results of this work, besides raising doubts about the use of traditional virulence markers of the species to predict the pathogenesis of a given *V. parahaemolyticus* strain, showed that reliable models are indispensable to evaluate the pathogenic potential of environmental isolates, classified as non-pathogenic based on the absence of the long-standing virulence indicators of the species. Therefore, *V. parahaemolyticus* unidentified factors that might be required to establish an infection, and screening assays in infection model hosts such as intraperitoneally injected mice and *G. mellonella* larvae would certainly help in the identification of novel virulence-related genes of the species.

The presence of potentially pathogenic *V. parahaemolyticus* in marine and estuarine environments and aquaculture settings in Brazil has been previously reported (Sobrinho et al., 2010; Rojas et al., 2011; Ramos et al., 2012).

Our findings corroborate these reports and strongly suggest that frequent environmental monitoring of aquaculture areas is essential to reduce the dissemination of pathogenic *V. parahaemolyticus* in seafood and avoid fatal cases of human infection induced by this species in the country.

## Data Availability Statement

The original contributions presented in the study are included in the article/Supplementary Material, further inquiries can be directed to the corresponding author. The datasets generated for this study can be found in the GenBank-accession numbers from MW254213 to MW254233.

## Ethics Statement

The animal study was reviewed and approved by the Comissão de Ética no Uso de Animais (CEUA) of the Centro de Ciências da Saúde (CCS) of the Universidade Federal do Rio de Janeiro (UFRJ).

## Author Contributions

LS, CL, and AA performed the experiments. LS, PB, and WK conceived and designed the study. LS, CL, PB, and WK analyzed the data. LS, CL, and WK wrote the manuscript. All authors contributed to the article and approved the submitted version.

## Conflict of Interest

The authors declare that the research was conducted in the absence of any commercial or financial relationships that could be construed as a potential conflict of interest.
