# Supplementary material for: Genotypic Diversity and Pathogenic Potential of Clinical and Environmental Vibrio parahaemolyticus Isolates From Brazil
Source: Front Microbiol. 2021 Mar 12;12:602653. doi: 10.3389/fmicb.2021.602653 (PMC7994283; doi:10.3389/fmicb.2021.602653)
Supplement: Supplementary file 1 [file Image_1.PDF]

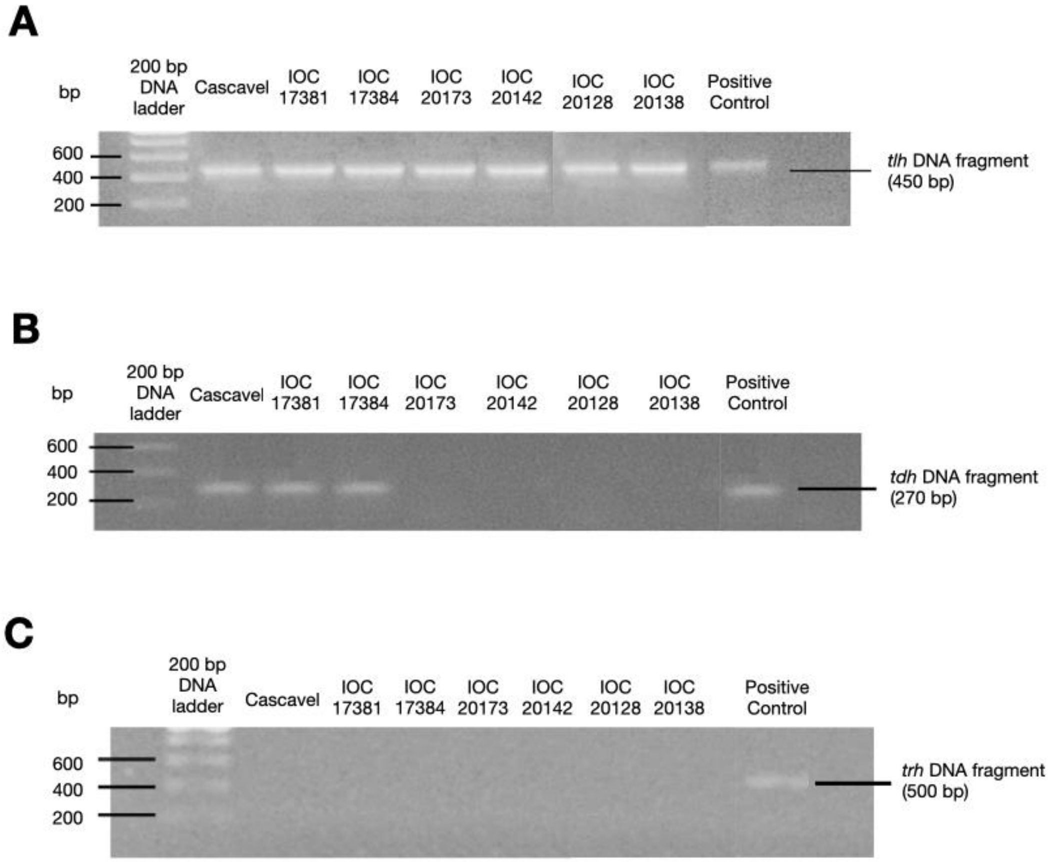

Supplementary Figure S1. PCR products containing the *tlh*, *tdh* and *trh* genes of *V. parahaemolyticus*. For the PCRs, samples of chromosomal DNA of the seven *V. parahaemolyticus* strains were used as templates with a pair of primers specific for each gene (Table 2), as recommended by FDA, 2004. The products were analyzed by electrophoresis on 1% agarose gel in TAE 1X and visualized by ethidium bromide staining. **(A)** PCR products containing the *tlh* gene (450bp). **(B)** PCR products containing the *tdh* gene (270 bp). **(C)** PCR products containing the *trh* gene (500bp). The positive control for genes *tlh* and *tdh* was the strain *V. parahaemolyticus* A431; the positive control for gene *trh* was *V. parahaemolyticus* strain 0798081. DNA marker: double stranded 200 bp ladder (Promega).
