# Supplementary material for: Genotypic Diversity and Pathogenic Potential of Clinical and Environmental Vibrio parahaemolyticus Isolates From Brazil
Source: Front Microbiol. 2021 Mar 12;12:602653. doi: 10.3389/fmicb.2021.602653 (PMC7994283; doi:10.3389/fmicb.2021.602653)
Supplement: Supplementary file 2 [file Image_2.PDF]

```
Vp_Cascavel     ATGTTTGACTCTATGATAAGCAAAAATACTTTTAATAAGATCGCAATGCTATTACTCCTC 60
Vp_17381        ATGTTTGACTCTATGATAAGCAAAAATACTTTTAATAAGATCGCAATGCTATTACTCCTC 60
Vp_17384        ATGTTTGACTCTATGATAAGCAAAAATACTTTTAATAAGATCGCAATGCTATTACTCCTC 60
Vp_20128        ATGTTTGACTCTATGATAAGCAAAAATACTTTTAATAAGATCGCAATGCTATTACTCCTC 60
Vp_20138        ATGTTTGACTCTATGATAAGCAAAAATACTTTTAATAAGATCGCAATGCTATTACTCCTC 60
Vp_20142        ATGTTTGACTCTATGATAAGCAAAAATACTTTTAATAAGATCGCAATGCTATTACTCCTC 60
Vp_20173        ATGTTTGACTCTATGATAAGCAAAAATACTTTTAATAAGATCGCAATGCTATTACTCCTC 60
Vp_RIMD2210633  ATGTTTGACTCTATGATAAGCAAAAATACTTTTAATAAGATCGCAATGCTATTACTCCTC 60
Vp_BB22OP       ATGTTTGACTCTATGATAAGCAAAAATACTTTTAATAAGATCGCAATGCTATTACTCCTC 60
                ************************************************************

Vp_Cascavel     ACGAGTTTGTTTGGTTGTAATAGCGACAGTGGAAGCAGCACTAGCCAACCACCAATGAGC 120
Vp_17381        ACGAGTTTGTTTGGTTGTAATAGCGACAGTGGAAGCAGCACTAGCCAACCACCAATGAGC 120
Vp_17384        ACGAGTTTGTTTGGTTGTAATAGCGACAGTGGAAGCAGCACTAGCCAACCACCAATGAGC 120
Vp_20128        ACGAGTTTGTTTGGTTGTAATAGCGACAGTGGAAGCAGCACTAGCCAACCACCAATGAGC 120
Vp_20138        ACGAGTTTGTTTGGTTGTAATAGCGACAGTGGAAGCAGCACTAGCCAACCACCAATGAGC 120
Vp_20142        ACGAGTTTGTTTGGTTGTAATAGCGACAGTGGAAGCAGCACTAGCCAACCACCAATGAGC 120
Vp_20173        ACGAGTTTGTTTGGTTGTAATAGCGACAGTGGAAGCAGCACTAGCCAACCACCAATGAGC 120
Vp_RIMD2210633  ACGAGTTTGTTTGGTTGTAATAGCGACAGTGGAAGCAGCACTAGCCAACCACCAATGAGC 120
Vp_BB22OP       ACGAGTTTGTTTGGTTGTAATAGCGACAGTGGAAGCAGCACTAGCCAACCACCAATGAGC 120
                ************************************************************

Vp_Cascavel     TTGCCAGAAGAAGTATTACCTGACGAGCCAGAAATTGAAGCACCAATACCGGAACTGCCA 180
Vp_17381        TTGCCAGAAGAAGTATTACCTGACGAGCCAGAAATTGAAGCACCAATACCGGAACTGCCA 180
Vp_17384        TTGCCAGAAGAAGTATTACCTGACGAGCCAGAAATTGAAGCACCAATACCGGAACTGCCA 180
Vp_20128        TTGCCAGAAGAAGTATTACCTGACGAGCCAGAAATTGAAGCGCCAATACCGGAACTGCCA 180
Vp_20138        TTGCCAGAAGAAGTATTACCTGACGAGCCAGAAATTGAAGCACCAATACCGGAACTGCCA 180
Vp_20142        TTGCCAGAAGAAGTATTACCTGACGAGCCAGAAAATGAAGCGCCAATACCGGAACTGCCA 180
Vp_20173        TTGCCAGAAGAAGTATTACCTGACGAGCCAGAAAATGAAGCGCCAATACCGGAACTGCCA 180
Vp_RIMD2210633  TTGCCAGAAGAAGTATTACCTGACGAGCCAGAAATTGAAGCACCAATACCGGAACTGCCA 180
Vp_BB22OP       TTGCCAGAAGAAGTATTACCTGACGAGCCAGAAATTGAAGCGCCAATACCGGAACTGCCA 180
                ********************************** ****** ******************

Vp_Cascavel     GTGCCTGAGTTTCCTGATATTTCCAAACCAGAAGTTCCTGAAACGCCAGAGGTGACGCCG 240
Vp_17381        GTGCCTGAGTTTCCTGATATTTCCAAACCAGAAGTTCCTGAAACGCCAGAGGTGACGCCG 240
Vp_17384        GTGCCTGAGTTTCCTGATATTTCCAAACCAGAAGTTCCTGAAACGCCAGAGGTGACGCCG 240
Vp_20128        GGGCCTGAGTTTCCTGATATTTCCAAACCAGAAGTTCCTGAAACGCCAGAGGTGACGCCG 240
Vp_20138        GTGCCTGAGTTTCCTGATATTTCCAAACCGGAAGTTCCTGAAACGCCAGAGGTGACGCCG 240
Vp_20142        GTGCCTGAGTTTCCTGATATTTCCAAACCGGAAGTTCCTGAAACGCCAGAGGTGACGCCG 240
Vp_20173        GTGCCTGAGTTTCCTGATATTTCCAAACCGGAAGTTCCTGAAACGCCAGAGGTGACGCCG 240
Vp_RIMD2210633  GTGCCTGAGTTTCCTGATATTTCCAAACCAGAAGTTCCTGAAACGCCAGAGGTGACGCCG 240
Vp_BB22OP       GTGCCTGAGTTTCCTGATATTTCCAAACCGGAAGTTCCTGAAACGCCAGAGGTGACGCCG 240
                * *************************** ******************************

Vp_Cascavel     CCGGAGCCTTCTGAACCCGTTATTCCGGAGCCGCCAGTACCAGACAAAGTGGTGTCTCGT 300
Vp_17381        CCGGAGCCTTCTGATCCCGTTATTCCCGAGCCGCCAGTACCAGACAAAGTGGTGTCTCGT 300
Vp_17384        CCGGAGCCTTCTGATCCCGTTATTCCCGAGCCGCCAGTACCAGACAAAGTGGTGTCTCGT 300
Vp_20128        CCGGAGCCTTCTGAACCCGTTATTCCGGAGCCGCCAGTACCAGACAAAGTGGTGTCTCGT 300
Vp_20138        CCGGAGCCTTCTGATCCCGTTATTCCAGAGCCGCCAGTACCAGACAAAGTGGTGTCTCGT 300
Vp_20142        CCGGAGCCTTCTGATCCCGTTATTCCCGAGCCGCCAGTACCAGATAAAGTGGTGTCTCGT 300
Vp_20173        CCGGAGCCTTCTGATCCCGTTATTCCCGAGCCGCCAGTACCAGATAAAGTGGTGTCTCGT 300
Vp_RIMD2210633  CCGGAGCCTTCTGATCCCGTTATTCCCGAGCCGCCAGTACCAGACAAAGTGGTGTCTCGT 300
Vp_BB22OP       CCGGAGCCTTCTGATCCCGTTATTCCCGAACCGCCAGTACCAGACAAAGTGGTGTCTCGT 300
                ************** *********** ** ************** ***************
```

```
Vp_Cascavel      ATTGCACTAAACCAAAGCCGTGTGTCGCTTGCAGAGGGTGAGCTGGCGCAGGTGAAAGTC 360
Vp_17381         ATTGCGCTAAACCAAAGCCGTGTGTCGCTTGCAGAGGGTGAGCTGGCACAGGTGAAAGTC 360
Vp_17384         ATTGCGCTAAACCAAAGCCGTGTGTCGCTTGCAGAGGGTGAGCTGGCACAGGTGAAAGTC 360
Vp_20128         ATTGCACTAAACCAAAGCCGTGTGTCGCTTGCAGAGGGTGAGCTGGCGCAGGTGAAAGTC 360
Vp_20138         ATTACACTAAACCAAAGCCGTGTGTCGCTTGCAGAGGGTGAGCTGGCACAGGTGAAAGTC 360
Vp_20142         ATTGCGCTAAACCAAAGCCGTGTGTCGCTTGCAGAAGGTGAGCTGGCGCAGGTGAAAGTC 360
Vp_20173         ATTGCGCTAAACCAAAGCCGTGTGTCGCTTGCAGAAGGTGAGCTGGCGCAGGTGAAAGTC 360
Vp_RIMD2210633   ATTGCGCTAAACCAAAGCCGTGTGTCGCTTGCAGAGGGTGAGCTGGCACAGGTGAAAGTC 360
Vp_BB22OP        ATTGCGCTAAACCAAAGCCGTGTGTCGCTTGCAGAAGGTGAGCTGGCGCAGGTGAAAGTC 360
                 *** * ***************************** *********** ************

Vp_Cascavel      ATCGGCTTTTATAACGATGGCACTCAAGACGACCTTACATCACAAGTTGTTTGGAACGTT 420
Vp_17381         ATCGGCTTTTATAACGATGACACTCAAGACGACCTTACATCACAAGTTGTTTGGAACGTT 420
Vp_17384         ATCGGCTTTTATAACGATGACACTCAAGACGACCTTACATCACAAGTTGTTTGGAACGTT 420
Vp_20128         ATCGGCTTTTATAACGATGGCACTCAAGACGACCTTACATCACAAGTTGTTTGGAACGTT 420
Vp_20138         ATCGGCTTTTATAACGATGACACTCAAGACGACCTTACATCACAAGTTGTTTGGAACGTT 420
Vp_20142         ATCGGCTTTTATAACGATGACACTCAAGACGACCTTACATCACAAGTTGTTTGGAACGTT 420
Vp_20173         ATCGGCTTTTATAACGATGACACTCAAGACGACCTTACATCACAAGTTGTTTGGAACGTT 420
Vp_RIMD2210633   ATCGGCTTTTATAACGATGACACTCAAGACGACCTTACATCACAAGTTGTTTGGAACGTT 420
Vp_BB22OP        ATCGGCTTTTATAACGATGACACTCAAGACGACCTTACATCACAAGTTGTTTGGAACGTT 420
                 ******************* ****************************************

Vp_Cascavel      GAAAATGCTGACATTGTTAACATCGACGAACGCGGTGTGATCACAGCGCTTAAAACAGGT 480
Vp_17381         GAAAATGCTGACATTGTTAACATCGACGAACGCGGTGTGATTACAGCGGTGAAAACGGGT 480
Vp_17384         GAAAATGCTGACATTGTTAACATCGACGAACGCGGTGTGATTACAGCGGTGAAAACGGGT 480
Vp_20128         GAAAATGCTGACATTGTTAACATCGACGAACGCGGTGTGATCACAGCGCTTAAAACAGGT 480
Vp_20138         GAAAATGCTGACATTGTTAACATCGACGAACGCGGTGTGATTACAGCGGTGAAAACGGGT 480
Vp_20142         GAAAATGCTGACATTGTTAACATCGACGAACGCGGTGTGATTACAGCGGTTAAAACGGGT 480
Vp_20173         GAAAATGCTGACATTGTTAACATCGACGAACGCGGTGTGATTACAGCGGTTAAAACGGGT 480
Vp_RIMD2210633   GAAAATGCTGACATTGTTAACATCGACGAACGCGGTGTGATTACAGCGGTGAAAACGGGT 480
Vp_BB22OP        GAAAATGCTGACATTGTTAACATCGACGAACGCGGTGTGATTACAGCGGTTAAAACGGGT 480
                 ***************************************** ****** * ***** ***

Vp_Cascavel      GTTTCGGTCATTCGCGCAGACCTAGATGAGTTCAGTAGCGAAATGACCGTACAGGTTGTG 540
Vp_17381         GTTTCGGTCATTCGCGCAGACCTAGATGAGTTCAGTAGCGAAATGACTGTACAGGTTGTG 540
Vp_17384         GTTTCGGTCATTCGCGCAGACCTAGATGAGTTCAGTAGCGAAATGACTGTACAGGTTGTG 540
Vp_20128         GTTTCGGTCGTTCGCGCAGACCTAGATGAGTTCAGTAGCGAAATGACCGTACAGGTTGTG 540
Vp_20138         GTTTCGGTCATTCGCGCAGACCTAGATGAGTTCAGTAGCGAAATGACTGTACAGGTTGTG 540
Vp_20142         GTTTCGGTCATTCGCGCAGACCTAGATGAGTTCAGTAGCGAAATGACTGTACAGGTTGTG 540
Vp_20173         GTTTCGGTCATTCGCGCAGACCTAGATGAGTTCAGTAGCGAAATGACTGTACAGGTTGTG 540
Vp_RIMD2210633   GTTTCGGTCATTCGCGCAGACCTAGATGAGTTCAGTAGCGAAATGACTGTACAGGTTGTG 540
Vp_BB22OP        GTTTCGGTCATTCGCGCAGACCTAGATGAGTTCAGTAGCGAAATGACTGTACAGGTTGTG 540
                 ********* ************************************* ************

Vp_Cascavel      GACGCGAAGTTGACCAACATTTACTTCGATCTGCCACATAAGGCGATTGCCAAAGGGCTA 600
Vp_17381         GACGCGAAGTTGACCAACATTTACTTCGATCTGCCACATAAGGCGATTGCCAAAGGGCTA 600
Vp_17384         GACGCGAAGTTGACCAACATTTACTTCGATCTGCCACATAAGGCGATTGCCAAAGGGCTA 600
Vp_20128         GACGCGAAGTTGACCAACATTTACTTCGATCTGCCACATAAGGCGATTGCCAAAGGGCTA 600
Vp_20138         GACGCGAAGTTGACCAACATTTACTTCGATCTGCCACATAAGGCGATTGCCAAAGGGCTA 600
Vp_20142         GACGCGAAGTTGACTAACATTTACTTCGATCTGCCACATAAGGCGATTGCCAAAGGGCTA 600
Vp_20173         GACGCGAAGTTGACTAACATTTACTTCGATCTGCCACATAAGGCGATTGCCAAAGGGCTA 600
Vp_RIMD2210633   GACGCGAAGTTGACCAACATTTACTTCGATCTGCCACATAAGGCGATTGCCAAAGGGCTA 600
Vp_BB22OP        GACGCGAAGTTGACTAACATTTACTTCGATCTGCCACATAAGGCGATTGCCAAAGGGCTA 600
                 ************** *********************************************
```

```
Vp_Cascavel      AGCTTTCAAGCGAAAGCTTACGGTGAATATACTGACAAACAAACCCGTGATATTAGCCAT 660
Vp_17381         AGCTTTCAAGCGAAAGCTTACGGTGAATATACCGACAAACAAACTCGTGATATTAGCCAT 660
Vp_17384         AGCTTTCAAGCGAAAGCTTACGGTGAATATACCGACAAACAAACTCGTGATATTAGCCAT 660
Vp_20128         AGCTTTCAAGCGAAAGCTTACGGTGAATATACTGACAAACAAACCCGTGATATTAGCCAT 660
Vp_20138         AGCTTTCAAGCGAAAGCTTACGGTGAATATACCGACAAACAAACCCGTGATATTAGCCAT 660
Vp_20142         AGCTTTCAAGCGAAAGCTTACGGTGAATATACCGACAAACAAACCCGTGATATTAGCCAT 660
Vp_20173         AGCTTTCAAGCGAAAGCTTACGGTGAATATACCGACAAACAAACCCGTGATATTAGCCAT 660
Vp_RIMD2210633   AGCTTTCAAGCGAAAGCTTACGGTGAATATACCGACAAACAAACTCGTGATATTAGCCAT 660
Vp_BB22OP        AGCTTTCAAGCGAAAGCTTACGGTGAATATACCGACAAACAAACCCGTGATATTAGCCAT 660
                 ******************************** *********** ***************

Vp_Cascavel      CTCGTAGAGTGGGATTCCACAGATTGTAGCGTTGTCATGCCAGGCACAAATGGCATTTTC 720
Vp_17381         CTCGTAGAGTGGGACTCCACAGATTGTAGCGTTGTCATGCCAGGCATAAATGGTATTTTC 720
Vp_17384         CTCGTAGAGTGGGACTCCACAGATTGTAGCGTTGTCATGCCAGGCATAAATGGTATTTTC 720
Vp_20128         CTCGTAGAGTGGGATTCCACAGATTGTAGCGTTGTCATGCCAGGCACAAATGGCATTTTC 720
Vp_20138         CTCGTAGAGTGGGATTCCACAGATTGTAGCGTTGTCATGCCAGGCACAAATGGCATTTTC 720
Vp_20142         CTCGTAGAGTGGGATTCCACAGATTGTAGCGTTGTCATACCAGGCACAAATGGCATTTTC 720
Vp_20173         CTCGTAGAGTGGGATTCCACAGATTGTAGCGTTGTCATACCAGGCACAAATGGCATTTTC 720
Vp_RIMD2210633   CTCGTAGAGTGGGACTCCACAGATTGTAGCGTTGTCATGCCAGGCATAAATGGTATTTTC 720
Vp_BB22OP        CTCGTAGAGTGGGACTCCACAGATTGTAGCGTCGTCACGCCAGGCATAAATGGTATTTTC 720
                 ************** ***************** ****  ******* ****** ******
Vp_Cascavel      ACCGCACAAGATGAGGGTGACGCGGATATCTATGCCGAGCTTGATGGCTTAACCAGTACG 780
Vp_17381         ACCGCACAAGATGAGGGTGACGCGGATATCTATGCCGAGCTTGATGGCTTAACCAGTACG 780
Vp_17384         ACCGCACAAGATGAGGGTGACGCGGATATCTATGCCGAGCTTGATGGCTTAACCAGTACG 780
Vp_20128         ACCGCACAAGATGAGGGTGACGCGGATATCTATGCCGAGCTTGATGGCTTAACCAGTACG 780
Vp_20138         ACCGCACAAGATGAGGGTGACGCGGATATCTATGCCGAGCTTGATGGCTTAACCAGTACG 780
Vp_20142         ACCGCACAAGATGAGGGTGACGCGGATATCTATGCCGAGCTTGATGGCTTAACCAGTACG 780
Vp_20173         ACCGCACAAGATGAGGGTGACGCGGATATCTATGCCGAGCTTGATGGCTTAACCAGTACG 780
Vp_RIMD2210633   ACCGCACAAGATGAGGGTGACGCGGATATCTATGCCGAGCTTGATGGCTTAACCAGTACG 780
Vp_BB22OP        ACCGCACAAGATGAGGGTGACGCGGATATTTATGCCGAGCTTGATGGCTTAACCAGTACG 780
                 ***************************** ******************************

Vp_Cascavel      CATGGTTCTATTACTGTCACGCCTGCGGTATTAGTGAGTATGGACATTTCGTCAACCGCA 840
Vp_17381         CATGGTTCTATTACTGTCACGCCTGCGGTATTAGTGAGTATGGACATTTCGTCAACCGCA 840
Vp_17384         CATGGTTCTATTACTGTCACGCCTGCGGTATTAGTGAGTATGGACATTTCGTCAACCGCA 840
Vp_20128         CATGGTTCTATTACTGTCACGCCTGCGGTATTAGTGAGTATGGACATTTCGTCAACCGCA 840
Vp_20138         CATGGTTCTATTACTGTCATGCCTGCGGTATTAGTGAGTATGGAGATTTCGTCAACCGCT 840
Vp_20142         CATGGTTCTATTACTGTCACGCCTGCGGTATTAGTGAGTATGGACATTTCGTCAACCGCA 840
Vp_20173         CATGGTTCTATTACTGTCACGCCTGCGGTATTAGTGAGTATGGACATTTCGTCAACCGCA 840
Vp_RIMD2210633   CATGGTTCTATTACTGTCACGCCTGCGGTATTAGTGAGTATGGACATTTCGTCAACCGCA 840
Vp_BB22OP        CATGGTTCTATTACTGTCACGCCTGCGGTATTAGTGAGTATGGACATTTCGTCAACCGCA 840
                 ******************* ************************ ************** 

Vp_Cascavel      CTGGAAATGCCGTTGGGCACACATAAGCCTTTGGTGGTCATGGGAACGTTGAGCGATGGT 900
Vp_17381         CTGGAAATGCCGTTGGGCACACATAAGCCTTTGGTGGTCATGGGAACGTTGAGCGATGGT 900
Vp_17384         CTGGAAATGCCGTTGGGCACACATAAGCCTTTGGTGGTCATGGGAACGTTGAGCGATGGT 900
Vp_20128         CTGGAAATGCCGTTGGGCACACATAAGCCTTTGGTGGTCATGGGAACGTTGAGCGATGGT 900
Vp_20138         CTGGAAATGCCGTTGGGCACACATAAGCCTTTGGTGGTCATGGGAACGTTGAGCGATGGT 900
Vp_20142         CTGGAAATGCCGTTGGGCACACATAAGCCTTTGGTGGTCATGGGAACGTTGAGCGATGGT 900
Vp_20173         CTGGAAATGCCGTTGGGCACACATAAGCCTTTGGTGGTCATGGGAACGTTGAGCGATGGT 900
Vp_RIMD2210633   CTGGAAATGCCGTTGGGCACACATAAGCCTTTGGTGGTCATGGGAACGTTGAGCGATGGT 900
Vp_BB22OP        CTGGAAATGCCGTTGGGCACACATAAGCCTTTGGTGGTCATGGGAACGTTGAGCGATGGT 900
                 ************************************************************
```

```
Vp_Cascavel      GAACAAGTCGACCTCACCCGAGGTATCACTTGGCACGTCGATAACGATGTGGTTGAGATT 960
Vp_17381         GAACAAGTCGACCTCACCCGAGGTATCACTTGGCACGTCGATAACGATGTGGTTGAGATT 960
Vp_17384         GAACAAGTCGACCTCACCCGAGGTATCACTTGGCACGTCGATAACGATGTGGTTGAGATT 960
Vp_20128         GAACAAGTCGACCTCACCCGAGGTATCACTTGGCACGTCGATAACGATGTGGTTGAGATT 960
Vp_20138         GAACAAGTCGACCTCACCCGAGGCATCACTTGGCACGCCGACAACGATGTGGTTGAGATT 960
Vp_20142         GAACAAGTCGACCTCTCCCGAGGTATCACTTGGCACGTCGATAACGATGTGGTTGAGATT 960
Vp_20173         GAACAAGTCGACCTCTCCCGAGGTATCACTTGGCACGTCGATAACGATGTGGTTGAGATT 960
Vp_RIMD2210633   GAACAAGTCGACCTCACCCGAGGTATCACTTGGCACGTCGATAACGATGTGGTTGAGATT 960
Vp_BB22OP        GAACAAGTCGACCTCACCCGAGGTATCACTTGGCACGTCGATAACGATGTGGTTGAGATT 960
                 *************** ******* ************* *** ******************

Vp_Cascavel      GTCGACAACGTTGTGAAAGCCAAACATAAAGGAACAGCGTTGGTGACGGCTGCGTCAGAT 1020
Vp_17381         GTCGACAACGTTGTGAAAGCCAAACATAAAGGAACAGCGTTGGTGACAGCTGCGTTAGAT 1020
Vp_17384         GTCGACAACGTTGTGAAAGCCAAACATAAAGGAACAGCGTTGGTGACAGCTGCGTTAGAT 1020
Vp_20128         GTCGACAACGTTGTGAAAGCCAAACATAAAGGAACAGCGTTGGTGACGGCTGCGTCAGAT 1020
Vp_20138         GTCGACAACGTTGTGAAAGCCAAACATAAAGGAACAGCGTTGGTGACGGCTGCGTTAGAT 1020
Vp_20142         GTCGACAACGTTGTGAAAGCCAAACATAAAGGAACAGCGTTGGTGACGGCTGCGTTAGAT 1020
Vp_20173         GTCGACAACGTTGTGAAAGCCAAACATAAAGGAACAGCGTTGGTGACGGCTGCGTTAGAT 1020
Vp_RIMD2210633   GTCGACAACGTTGTGAAAGCCAAACATAAAGGAACAGCGTTGGTGACAGCTGCGTTAGAT 1020
Vp_BB22OP        GTCGACAACGTTGTGAAAGCCAAACATAAAGGAACAGCGTTGGTGACGGCTGCGTTAGAT 1020
                 *********************************************** ******* ****

Vp_Cascavel      GGTGTGCAAAGCGAGCCAATACAAGTGCAGGTAACCGATGCAATCCTAACAAATATTGAA 1080
Vp_17381         GGTGTGCAAAGCGAGCCAATACAAGTGCAGGTAAACGATGCAATCCTAACAAATATTGAA 1080
Vp_17384         GGTGTGCAAAGCGAGCCAATACAAGTGCAGGTAAACGATGCAATCCTAACAAATATTGAA 1080
Vp_20128         GGTGTGCAAAGCGAGCCAATACAAGTGCAGGTAACCGATGCAATCCTAACAAATATTGAA 1080
Vp_20138         GGTGTGCAAAGCGAGCCAATACAAGTGCAGGTGACCGATGCAATCCTAACAAATATTGAA 1080
Vp_20142         GGTGTGCAAAGCGAGCCAATACAAGTGCAGGTGACCGATGCAATCCTAACAAATATTGAA 1080
Vp_20173         GGTGTGCAAAGCGAGCCAATACAAGTGCAGGTGACCGATGCAATCCTAACAAATATTGAA 1080
Vp_RIMD2210633   GGTGTGCAAAGCGAGCCAATACAAGTGCAGGTAAACGATGCAATCCTAACAAATATTGAA 1080
Vp_BB22OP        GGTGTGCAAAGCGAGCCAATACAAGTGCAGGTGACCGATGCAATCCTAACAAATATTGAA 1080
                 ******************************** * *************************
Vp_Cascavel      GTGACAACGAATGCGTCGAGCGTGGCAAAAGGCAACTCGACCCTGCTGTCTGCGCAAGGA 1140
Vp_17381         GTGACAACGAATGCGTCGAGCGTGGCAAAAGGCAACTCGACCCTGCTGTCTGCACAAGGA 1140
Vp_17384         GTGACAACGAATGCGTCGAGCGTGGCAAAAGGCAACTCGACCCTGCTGTCTGCACAAGGA 1140
Vp_20128         GTGACAACGAATGCGTCGAGCGTGGCAAAAGGCAACTCGAGCCTGCTGTCTGCACAAGGA 1140
Vp_20138         GTGACAACGAATGCGTCGAGCGTGGCAAAAGGCAACTCGACCCAGCTGTCTGCACAAGGA 1140
Vp_20142         GTGACAACGAATGCGTCGAGCGTGGCAAAAGGCAACTCGACCCTGCTGTCTGCACAAGGA 1140
Vp_20173         GTGACAACGAATGCGTCGAGCGTGGCAAAAGGCAACTCGACCCTGCTGTCTGCACAAGGA 1140
Vp_RIMD2210633   GTGACAACGAATGCGTCGAGCGTGGCAAAAGGCAACTCGACCCTGCTGTCTGCACAAGGA 1140
Vp_BB22OP        GTGACAACGAATGCGTCGAGCGTGGCAAAAGGCAACTCGACCCAGCTGTCTGCACAAGGA 1140
                 **************************************** ** ********* ******

Vp_Cascavel      GTTTATAGCGATGAAAGCCGCGTTGAGTTGACCGAACAGGTTGCTTGGTGGGTTGATAAT 1200
Vp_17381         GTCTATAGCGATGAAAGCCGCGTTGAGTTGACCGAACAGGTTGCTTGGTGGGTTGATAAT 1200
Vp_17384         GTCTATAGCGATGAAAGCCGCGTTGAGTTGACCGAACAGGTTGCTTGGTGGGTTGATAAT 1200
Vp_20128         GTTTATAGCGATGAAAGCCGCGTTGAGTTGACCGAACAGGTTGCTTGGTGGGTTGATAAT 1200
Vp_20138         GTTTATAGCGATGAAAGCCGCGTTGAGTTGACCGAACAGGTTGCTTGGTGGGTTGATAAT 1200
Vp_20142         GTTTATAGCGATGAAAGCCGCGTTGAGTTGACCGAACAGGTTGCTTGGTGGGTTGATAAT 1200
Vp_20173         GTTTATAGCGATGAAAGCCGCGTTGAGTTGACCGAACAGGTTGCTTGGTGGGTTGATAAT 1200
Vp_RIMD2210633   GTCTATAGCGATGAAAGCCGCGTTGAGTTGACCGAACAGGTTGCTTGGTGGGTTGATAAT 1200
Vp_BB22OP        GTTTATAGCGATGAAAGCCGCGTTGAGTTGACCGAACAGGTTGCTTGGTGGGTTGATAAT 1200
                 ** *********************************************************
```

```
Vp_Cascavel      CAAGATGTTCTACAACTAGAAGGCGCTCGCGTTAAAGGTCTTGCGGTAGGGCAGGCAATG 1260
Vp_17381         CAAGATGTCCTACAACTAGAAGGCGATCGCGTTAAAGGTCTTGCGGTAGGGCAGGCAATG 1260
Vp_17384         CAAGATGTCCTACAACTAGAAGGCGATCGCGTTAAAGGTCTTGCGGTAGGGCAGGCAATG 1260
Vp_20128         CAAGATGTCCTACAACTAGAAGGCGCTCGCGTTAAAGGTCTTGCGGTAGGGCAGGCAATG 1260
Vp_20138         CAAGATGTCCTACAATTAGAAGGCGCTCGCGTTAAAGGTCTTGCGGTAGGGCAGGCAATG 1260
Vp_20142         CAAGATGTCCTACAACTAGAAGGCGATCGCGTTAAAGGTCTTGCGGTAGGGCAGGCAATG 1260
Vp_20173         CAAGATGTCCTACAACTAGAAGGCGATCGCGTTAAAGGTCTTGCGGTAGGGCAGGCAATG 1260
Vp_RIMD2210633   CAAGATGTCCTACAACTAGAAGGCGATCGCGTTAAAGGTCTTGCGGTAGGGCAGGCAATG 1260
Vp_BB22OP        CAAGATGTTCTACAACTAGAAGGCGCTCGCGTTAAAGGTCTTGCGGTAGGGCAGGCGATG 1260
                 ******** ****** ********* ****************************** ***

Vp_Cascavel      GTTTACGCCACCAAAGATGCGATCACCAGTGCACCATTGCAAATTCAAGTAACGAATGCG 1320
Vp_17381         ATTTACGCCACCAAAGATGCGATCACCAGTGCACCATTGCAAATTCAAGTAACGAATGCG 1320
Vp_17384         ATTTACGCCACCAAAGATGCGATCACCAGTGCACCATTGCAAATTCAAGTAACGAATGCG 1320
Vp_20128         ATTTACGCCACCAAAGATGCGATCACCAGTGCACCATTGCAAATTCAAGTAACGAATGCG 1320
Vp_20138         GTTTACGCCACCAAAGATGCGATCACCAGTGCACCATTGCAAATTCAAGTAACGAATGCG 1320
Vp_20142         GTTTACGCCACCAAAGATGCGATCACCAGTGCACCATTGCAAATTCAAGTAACGAATGCG 1320
Vp_20173         GTTTACGCCACCAAAGATGCGATCACCAGTGCACCATTGCAAATTCAAGTAACGAATGCG 1320
Vp_RIMD2210633   ATTTACGCCACCAAAGATGCGATCACCAGTGCACCATTGCAAATTCAAGTAACGAATGCG 1320
Vp_BB22OP        ATTTACGCCACCAAAGATGCGATCACCAGTGCACCATTGCAAATTCAAGTAACGAATGCG 1320
                  ***********************************************************

Vp_Cascavel      GTGTTAGAAAAAATCCATGTTCATCCTAGTGAAATCACTCTAGAGGAAAAGAACGTCCAG 1380
Vp_17381         GTGTTAGAAAAAATCCATGTTCATCCTAGTGAAATCACTCTAGAGGAAAAGAACGTCCAG 1380
Vp_17384         GTGTTAGAAAAAATCCATGTTCATCCTAGTGAAATCACTCTAGAGGAAAAGAACGTCCAG 1380
Vp_20128         GTGTTAGAAAAAATCCATGTTCATCCTAGTGAAATCACTCTAGAGGAAAAGAACGTCCAG 1380
Vp_20138         GTGTTAGAAAAAATCCATGTTCATCCTAGTGAAATCACTCTAGAGGAAAAGAACGTCCAG 1380
Vp_20142         GTGTTAGAAAAAATCCATGTTCATCCTAGTGAAATCACTCTAGAGGAAAAGAACGTCCAG 1380
Vp_20173         GTGTTAGAAAAAATCCATGTTCATCCTAGTGAAATCACTCTAGAGGAAAAGAACGTCCAG 1380
Vp_RIMD2210633   GTGTTAGAAAAAATCCATGTTCATCCTAGTGAAATCACTCTAGAGGAAAAGAACGTCCAG 1380
Vp_BB22OP        GTGTTAGAAAAAATCCATGTTCATCCTAGTGAAATCACTCTAGAGGAAAAGAACGTCCAG 1380
                 ************************************************************

Vp_Cascavel      CGATTTTATGCCTACGGTGAATATAGCGATGAAACCACTCAGGATGTAACGCATCGAGTG 1440
Vp_17381         CGATTTTATGCCTACGGTGAATATAGCGATGAAACCACTCAGGATGTAACGCATCGAGTG 1440
Vp_17384         CGATTTTATGCCTACGGTGAATATAGCGATGAAACCACTCAGGATGTAACGCATCGAGTG 1440
Vp_20128         CGATTCTATGCCTACGGTGAATATAGCGATGAAACCACTCAGGATGTAACGCATCGAGTG 1440
Vp_20138         CGATTTTATGCCTACGGTGAATATAGCGATGAAACCACTCAGGATGTAACGCATCGAGTG 1440
Vp_20142         CGATTTTATGCCTACGGTGAATATAGCGATGAAACCACTCAGGATGTAACGCATCGAGTG 1440
Vp_20173         CGATTTTATGCCTACGGTGAATATAGCGATGAAACCACTCAGGATGTAACGCATCGAGTG 1440
Vp_RIMD2210633   CGATTTTATGCCTACGGTGAATATAGCGATGAAACCACTCAGGATGTAACGCATCGAGTG 1440
Vp_BB22OP        CGATTTTATGCCTACGGTGAATATAGCGATGAAACCACTCAGGATGTAACGCATCGAGTG 1440
                 ***** ******************************************************
Vp_Cascavel      ACATGGCGAAGCAGTAATAAAGCGGTTTTAGATTTGATTGCTGGTGGACTCTCAAATAGT 1500
Vp_17381         ACATGGCGAAGCAGTAATAAAGCGGTTTTAGATTTGATTGCTGGTGGGCTCTCAAATAGT 1500
Vp_17384         ACATGGCGAAGCAGTAATAAAGCGGTTTTAGATTTGATTGCTGGTGGGCTCTCAAATAGT 1500
Vp_20128         ACATGGCGAAGCAGTAATAAAGCGGTTTTAGATTTGATTGCTGGTGGACTCTCAAATAGT 1500
Vp_20138         ACATGGCGAAGCAGTAATAAAGCGGTTTTAGATTTGATTGCTGATGGACTCTCAAATAGT 1500
Vp_20142         ACATGGCGAAGCAGTAATAAAGCGGTTTTAGATTTGATTGCTGGTGGGCTCTCAAATAGT 1500
Vp_20173         ACATGGCGAAGCAGTAATAAAGCGGTTTTAGATTTGATTGCTGGTGGGCTCTCAAATAGT 1500
Vp_RIMD2210633   ACATGGCGAAGCAGTAATAAAGCGGTTTTAGATTTGATTGCTGGTGGGCTCTCAAATAGT 1500
Vp_BB22OP        ACATGGCGAAGCAGTAATAAAGCGGTTTTAGATTTGATTGCTGGTGGACTCTCAAATAGT 1500
                 ******************************************* *** ************
```

```
Vp_Cascavel     GCTCAGCTTGGTTCGGTCACCATTTCAGCTTCCTTTGGGGAGTTAAAAGGCATCTCTGAG 1560
Vp_17381        GCTCAGCTTGGTTCGGTCACCATTTCAGCTTCCCTTGGGGAGTTAAAAGGCACCTCTGAG 1560
Vp_17384        GCTCAGCTTGGTTCGGTCACCATTTCAGCTTCCCTTGGGGAGTTAAAAGGCACCTCTGAG 1560
Vp_20128        GCTCAGCTTGGTTCGGTCACCATTTCAGCGTCCCTCGGGGAGTTAAAAGGCATCTCTGAG 1560
Vp_20138        GCTCAGCTTGGTTCGGTTACCATTTCAGCTTCCCTTGGGGAGTTAAAAGGCATCTCTGAG 1560
Vp_20142        GCTCAGCTTGGTTCGGTCACCATTTCAGCTTCCCTTGGGGAGTTAAAAGGCACCTCTGAG 1560
Vp_20173        GCTCAGCTTGGTTCGGTCACCATTTCAGCTTCCCTTGGGGAGTTAAAAGGCACCTCTGAG 1560
Vp_RIMD2210633  GCTCAGCTTGGTTCGGTCACCATTTCAGCTTCCCTTGGGGAGTTAAAAGGCACCTCTGAG 1560
Vp_BB22OP       GCTCAGCTTGGTTCGGTCACCATTTCAGCTTCCTTTGGGGAGTTAAAAGGCATCTCTGAG 1560
                ***************** *********** *** * **************** *******

Vp_Cascavel     GTCAAGGTTGTAGAGGCGGAAGAAAAAGAGTCTGCCATCTCTATTTGCCCACAACCTCGT 1620
Vp_17381        GTCAAGGTTGTTGAGTCGGAAGAAAAAGAGTCTGCCATCTCTATATGCCCACAACCTCGT 1620
Vp_17384        GTCAAGGTTGTTGAGTCGGAAGAAAAAGAGTCTGCCATCTCTATATGCCCACAACCTCGT 1620
Vp_20128        GTCAAGGTTGTTGAGGCGGAAGAAAAAGAGTCTGCCATCTCTATTTGCCCACAACCTCGT 1620
Vp_20138        GTCAAGGTTGTTGAGGCGGAAGAAAAAGAGTCTGCCATCTCTATTTGCCCACAGCCTCGT 1620
Vp_20142        GTCAAGGTTGTTGAGGCGGAAGAAAAAGAGGCTGCCATCTCTATATGCCCACAACCTCGT 1620
Vp_20173        GTCAAGGTTGTTGAGGCGGAAGAAAAAGAGGCTGCCATCTCTATATGCCCACAACCTCGT 1620
Vp_RIMD2210633  GTCAAGGTTGTTGAGTCGGAAGAAAAAGAGTCTGCCATCTCTATATGCCCACAACCTCGT 1620
Vp_BB22OP       GTCAAGGTTGTAGAGGCGGAAGAAAAAGAGTCTGCCATCTCTATTTGCCCACAACCTCGT 1620
                *********** *** ************** ************* ******** ******

Vp_Cascavel     TCAGGAGAAGGAAGCGCGGAAGCGTGTTTGATGGTAGCGAGTGATGAAGATGGTCGCCTA 1680
Vp_17381        TCAGGAGAAGGAAGCGTGGAAGCGTGTTTGATGGTAGCGAGTGATGAAGATGGTCGCCTA 1680
Vp_17384        TCAGGAGAAGGAAGCGTGGAAGCGTGTTTGATGGTAGCGAGTGATGAAGATGGTCGCCTA 1680
Vp_20128        TCAGGAGAAGGACGCGCGGAAGCGTGTTTGATGGTAGCGAGTGATGAAGATGGTCGCCTA 1680
Vp_20138        TCAGGAGAAGGAAGCGCGGAAGCGTGTTTGATGGTAGCGAGTGATGAAGATGGTCGCCTA 1680
Vp_20142        TCAGGAGAAGGAAGCGTGGAAGCGTGTTTGATGGTAGCGAGTGATGAAGATGGTCGCCTA 1680
Vp_20173        TCAGGAGAAGGAAGCGTGGAAGCGTGTTTGATGGTAGCGAGTGATGAAGATGGTCGCCTA 1680
Vp_RIMD2210633  TCAGGAGAAGGAAGCGTGGAAGCGTGTTTGATGGTAGCGAGTGATGAAGATGGTCGCCTA 1680
Vp_BB22OP       TCAGGAGAAGGAAGCGCGGAAGCGTGTTTGATGGTAGCGAGTGATGAAGATGGTCGCCTA 1680
                ************ *** *******************************************

Vp_Cascavel     TTTACCGCACCACCATCAGTGAAGCTCATGAACAAACTAGGCTATGAGCTTGTTAAACCT 1740
Vp_17381        TTTACCGCACCACCATCAGTGAAGCTCATGAACAAACTAGGCTATGAGCTTGTTAAACCT 1740
Vp_17384        TTTACCGCACCACCATCAGTGAAGCTCATGAACAAACTAGGCTATGAGCTTGTTAAACCT 1740
Vp_20128        TTTACCGCACCACCATCAGTGAAGCTCATGAACAAACTAGGCTATGAGCTTGTTAAACCT 1740
Vp_20138        TTTACCGCACCACCATCAGTGAAGCTCATGAACAAACTAGGCTATGAGCTTGTTAAACCT 1740
Vp_20142        TTTACCGCACCACCATCAGTGAAGCTCATGAACAAACTAGGCTATGAGCTTGTTAAACCT 1740
Vp_20173        TTTACCGCACCACCATCAGTGAAGCTCATGAACAAACTAGGCTATGAGCTTGTTAAACCT 1740
Vp_RIMD2210633  TTTACCGCACCACCATCAGTGAAGCTCATGAACAAACTAGGCTATGAGCTTGTTAAACCT 1740
Vp_BB22OP       TTTACCGCACCACCATCAGTGAAGCTCATGAACAAACTAGGCTATGAGCTTGTTAAACCT 1740
                ************************************************************

Vp_Cascavel     TTGAGCTACGACGCGACAAAACCAAAAACTTACGTTGGCATCAAAACGGAAGACGGTACA 1800
Vp_17381        TTGAGCTACGACGCGACAAAACCAAAAACTTACGTTGGCATCAAAACGGAAGACGGTACA 1800
Vp_17384        TTGAGCTACGACGCGACAAAACCAAAAACTTACGTTGGCATCAAAACGGAAGACGGTACA 1800
Vp_20128        TTGAGCTACGACGCGACAAAACCGAAAACGTACGTTGGCATCAAAACGGAAGACGGTACA 1800
Vp_20138        TTGAGCTACGACGCGACAAAACCAAAAACTTACGTTGGCATCAAAACGGAAGACGGTACA 1800
Vp_20142        TTGAGCTACGACGCGACAAAACCAAAAACTTACGTTGGCATCAAAACGGAAGACGGTACA 1800
Vp_20173        TTGAGCTACGACGCGACAAAACCAAAAACTTACGTTGGCATCAAAACGGAAGACGGTACA 1800
Vp_RIMD2210633  TTGAGCTACGACGCGACAAAACCAAAAACTTACGTTGGCATCAAAACGGAAGACGGTACA 1800
Vp_BB22OP       TTGAGCTACGACGCGACAAAACCAAAAACTTACGTTGGCATCAAAACGGAAGACGGTACA 1800
                *********************** ***** ******************************
```

```
Vp_Cascavel      CGCGGCCCTGAGGGTGACTTTGCGATGTTCAACCAATACGGACGTGATCGCGCAGGCTTA 1860
Vp_17381         CGCGGCCCTGTGGGTGACTTTGCGATGTTCAACCAATACGGACGTGATCGCGCAGGCTTA 1860
Vp_17384         CGCGGCCCTGTGGGTGACTTTGCGATGTTCAACCAATACGGACGTGATCGCGCAGGCTTA 1860
Vp_20128         CGCGGCCCTGTGGGTGACTTTGCGATGTTCAACCAATACGGACGTGATCGCGCAGGCTTA 1860
Vp_20138         CGCGGCCCTGTGGGTGACTTTGCGATGTTCAACCAATACGGACGTGATCGCGCAGGCTTA 1860
Vp_20142         CGCGGCCCTGTGGGTGACTTTGCGATGTTCAACCAATACGGACGTGATCGCGCAGGCTTA 1860
Vp_20173         CGCGGCCCTGTGGGTGACTTTGCGATGTTCAACCAATACGGACGTGATCGCGCAGGCTTA 1860
Vp_RIMD2210633   CGCGGCCCTGTGGGTGACTTTGCGATGTTCAACCAATACGGACGTGATCGCGCAGGCTTA 1860
Vp_BB22OP        CGCGGCCCTGTGGGTGACTTTGCGATGTTCAACCAATACGGACGTGATCGCGCAGGCTTA 1860
                 ********** *************************************************

Vp_Cascavel      ACCAGCCAATACGCAAACTGGTGTCATGATTTGTCTGTAAGAAACTTTGCTGGCCGTGAC 1920
Vp_17381         ACCAGCCAATACGCAAACTGGTGTCATGATTTGTCTGTAAGAAACTTTGCTGGCCGTGAC 1920
Vp_17384         ACCAGCCAATACGCAAACTGGTGTCATGATTTGTCTGTAAGAAACTTTGCTGGCCGTGAC 1920
Vp_20128         ACCAGCCAATACGCAAACTGGTGCCATGATTTGTCTGTAAGAAACTTTGCTGGTCGTGAC 1920
Vp_20138         ACCAGCCAATACGCAAACTGGTGTCATGATTTGTCTGTAAGAAACTTTGCTGGCCGTGAC 1920
Vp_20142         ACCAGCCAATACGCAAACTGGTGTCATGATTTGTCTGTAAGAAACTTTGCTGGCCGTGAC 1920
Vp_20173         ACCAGCCAATACGCAAACTGGTGTCATGATTTGTCTGTAAGAAACTTTGCTGGCCGTGAC 1920
Vp_RIMD2210633   ACCAGCCAATACGCAAACTGGTGTCATGATTTGTCTGTAAGAAACTTTGCTGGCCGTGAC 1920
Vp_BB22OP        ACCAGCCAATACGCAAACTGGTGTCATGATTTGTCTGTAAGAAACTTTGCTGGCCGTGAC 1920
                 *********************** ***************************** ******

Vp_Cascavel      AACTGGCGTCGTGCCACTCGCAATGAGTTGTTTAGCTTGTATCGTGCATCACGAGGCAGT 1980
Vp_17381         AACTGGCGTCGTGCCACTCGCAATGAGTTGTTTAGCTTGTATCGTGCATCACGAGGCAGT 1980
Vp_17384         AACTGGCGTCGTGCCACTCGCAATGAGTTGTTTAGCTTGTATCGTGCATCACGAGGCAGT 1980
Vp_20128         AACTGGCGTCGTGCCACTCGCAATGAGTTGTTTAGCTTGTATCGTGCATCACGAGGCAGT 1980
Vp_20138         AATTGGCGTCGTGCCACTCGCAATGAGTTGTTTAGCTTGTATCGTGCATCACGAGGCAGT 1980
Vp_20142         AATTGGCGTCGTGCCACTCGCAATGAGTTGTTTAGCTTGTATCGTGCATCACGAGGCAGT 1980
Vp_20173         AATTGGCGTCGTGCCACTCGCAATGAGTTGTTTAGCTTGTATCGTGCATCACGAGGCAGT 1980
Vp_RIMD2210633   AACTGGCGTCGTGCCACTCGCAATGAGTTGTTTAGCTTGTATCGTGCATCACGAGGCAGT 1980
Vp_BB22OP        AATTGGCGTCGTGCCACTCGCAATGAGTTGTTTAGCTTGTATCGTGCATCACGAGGCAGT 1980
                 ** *********************************************************

Vp_Cascavel      GTTTGGGACGGGACTGAAAGCATCTATGAAGAAGACAAAGATGGCGGAGGTTTTGGTTGG 2040
Vp_17381         GTTTGGGACGGGACTGAAAGCATCTATGAAGAAGACAAAGATGGCGGAGGTTTTGGTTGG 2040
Vp_17384         GTTTGGGACGGGACTGAAAGCATCTATGAAGAAGACAAAGATGGCGGAGGTTTTGGTTGG 2040
Vp_20128         GTTTGGGACGGGACTGAAAGCATCTATGAAGAAGACAAAGATGGCGGAGGTTTTGGTTGG 2040
Vp_20138         GTTTGGGACGGGACTGAAAGCATCTATGAAGAAGACAAAGATGGCGGAGGTTTTGGTTGG 2040
Vp_20142         GTTTGGGACGGGACTGAAAGCATCTATGAAGAAGACAAAGATGGCGGAGGTTTTGGTTGG 2040
Vp_20173         GTTTGGGACGGGACTGAAAGCATCTATGAAGAAGACAAAGATGGCGGAGGTTTTGGTTGG 2040
Vp_RIMD2210633   GTTTGGGACGGGACTGAAAGCATCTATGAAGAAGACAAAGATGGCGGAGGTTTTGGTTGG 2040
Vp_BB22OP        GTTTGGGACGGGACTGAAAGCATCTATGAAGAAGACAAAGATGGCGGAGGTTTTGGTTGG 2040
                 ************************************************************

Vp_Cascavel      CCAGCGAACTCGGAATATTGGTCTACCTCGCTAATGGATTTACCGCATCATGAGGGTGTC 2100
Vp_17381         CCAGCGAACTCGGAATATTGGTCTACCTCGCTAATGGATTTACCGCATCATGAGGGCGTC 2100
Vp_17384         CCAGCGAACTCGGAATATTGGTCTACCTCGCTAATGGATTTACCGCATCATGAGGGCGTC 2100
Vp_20128         CCAGCGAACTCGGAATATTGGTCTACTTCGCTAATGGATTTACCGCATCATGAGGGCGTC 2100
Vp_20138         CCAGCGAATTCGGAATATTGGTCTACCTCGCTAATGGATTTACCGCATCACGAGGGCGTC 2100
Vp_20142         CCAGCGAATTCGGAATATTGGTCTACCTCGCTAATGGATTTACCGCATCACGAGGGCGTC 2100
Vp_20173         CCAGCGAATTCGGAATATTGGTCTACCTCGCTAATGGATTTACCGCATCACGAGGGCGTC 2100
Vp_RIMD2210633   CCAGCGAACTCGGAATATTGGTCTACCTCGCTAATGGATTTACCGCATCATGAGGGCGTC 2100
Vp_BB22OP        CCAGCGAACTCGGAATATTGGTCTACCTCGCTAATGGATTTACCGCATCATGAGGGCGTC 2100
                 ******** ***************** *********************** ***** ***
```

```
Vp_Cascavel     GTGTATGACATCATTAACCTGCATCGAGGTCGAGCGCAGTTGGTGATGGATGCTCGAGAC 2160
Vp_17381        GTGTATGACATCATTAACCTGCATCGAGGTCGAGCGCAGTTGGTGATGGATGCTCGAGAC 2160
Vp_17384        GTGTATGACATCATTAACCTGCATCGAGGTCGAGCGCAGTTGGTGATGGATGCTCGAGAC 2160
Vp_20128        GTGTATGACATCATTAACCTGCATCGAGGTCGAGCGCAGTTGGTGATGGATGCTCGAGAC 2160
Vp_20138        GTGTATGACATCATTAACCTGCATCGAGGTCGAGCGCAGTTGGTGATGGATGCTCGAGAC 2160
Vp_20142        GTGTATGACATCATTAACCTGCATCGAGGTCGAGCGCAGTTGGTGATGGATGCTCGAGAC 2160
Vp_20173        GTGTATGACATCATTAACCTGCATCGAGGTCGAGCGCAGTTGGTGATGGATGCTCGAGAC 2160
Vp_RIMD2210633  GTGTATGACATCATTAACCTGCATCGAGGTCGAGCGCAGTTGGTGATGGATGCTCGAGAC 2160
Vp_BB22OP       GTGTATGACATCATTAACCTGCATCGAGGTCGAGCGCAGTTGGTGATGGATGCTCGAGAC 2160
                ************************************************************

Vp_Cascavel     CCGGCTTATGCTTCCTGCGTCTCTGACGCGCCTGGCGTTCCTCTTAAGTAA 2211
Vp_17381        CCGGCTTATGCTTCCTGCGTGTCTGACGCGCCTGGCGTTCCTCTTAAGTAA 2211
Vp_17384        CCGGCTTATGCTTCCTGCGTGTCTGACGCGCCTGGCGTTCCTCTTAAGTAA 2211
Vp_20128        CTGGCTTATGCTTCCTGCGTGTCTGACGCGCCTGGCGTTCCTCTTAAGTAA 2211
Vp_20138        CCGGCTTATGCTTCCTGCGTGTCTGACGCGCCTGGCGTTCCTCTTAAGTAA 2211
Vp_20142        CCGGCTTATGCTTCCTGCGTGTCTGACGCGCCTGGCGTTCCTCTTAAGTAA 2211
Vp_20173        CCGGCTTATGCTTCCTGCGTGTCTGACGCGCCTGGCGTTCCTCTTAAGTAA 2211
Vp_RIMD2210633  CCGGCTTATGCTTCCTGCGTGTCTGACGCGCCTGGCGTTCCTCTTAAGTAA 2211
Vp_BB22OP       CCGGCTTATGCTTCCTGCGTGTCTGACGCGCCTGGCGTTCCTCTTAAGTAA 2211
                * ****************** ******************************
```

Supplementary Figure S2. Multiple alignment of *vp1767* gene sequences from the seven *V. parahaemolyticus* strains (Cascavel, IOC 17381, IOC 17384, IOC 20128, IOC 20138, IOC 20142 and IOC 20173). DNA fragments containing the *vp1767* gene (2,211 bp) from the seven *V. parahaemolyticus* strains, were sequenced (ABI PRISM® BigDye® Terminator v1.1 Ready Reaction Cycle Sequencing Kit; Applied Biosystems) and aligned with Clustal Omega program (Sievers et al., 2011; Larkin et al., 2007). The *vpadF* gene sequences from the reference strains RIMD2210633 (GenBank accession numbers BA000031 and BA000032 (Makino et al., 2003), and BB22OP (Genbank accession numbers CP003972 and CP003973) (Jensen et al., 2013) were also included in the alignment. Bases that vary among the sequences are shown in bold.
