# Supplementary material for: Genotypic Diversity and Pathogenic Potential of Clinical and Environmental Vibrio parahaemolyticus Isolates From Brazil
Source: Front Microbiol. 2021 Mar 12;12:602653. doi: 10.3389/fmicb.2021.602653 (PMC7994283; doi:10.3389/fmicb.2021.602653)
Supplement: Supplementary file 3 [file Image_3.pdf]

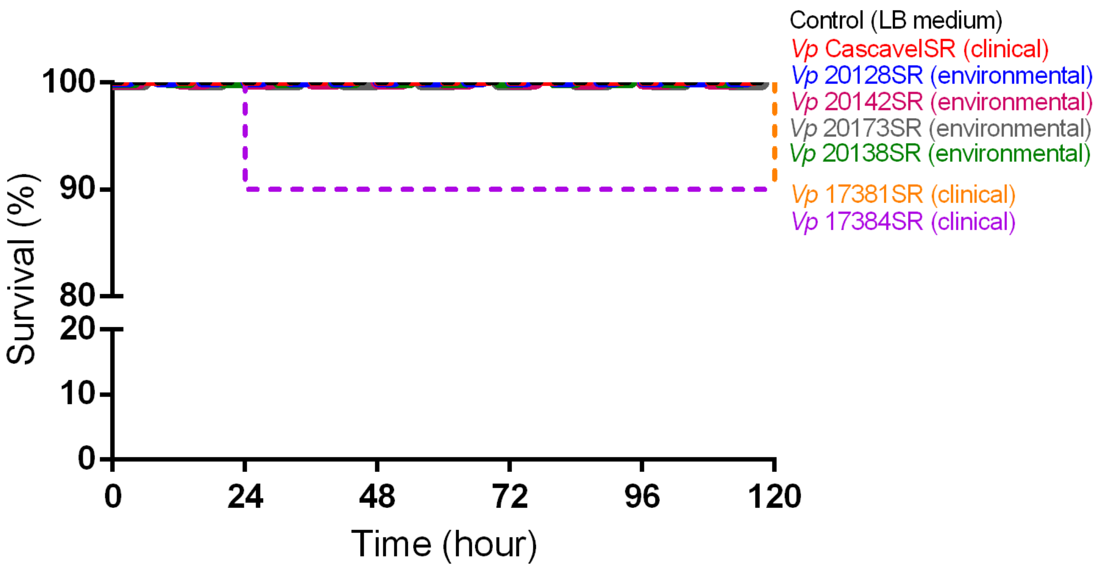

Supplementary Figure S3. Survival curves of mice inoculated with 10^6 CFU/animal of each of seven *V. parahaemolyticus* strains. Each test group contained n = 10 animals per strain. Control group: mice inoculated with LB medium, n = 5 animals. Survival curves of mice in each group were generated using the Kaplan-Meier method (Kaplan and Meier, 1958) and compared using the log-rank test (Mantel, 1966; Peto and Peto, 1972), with p-values < 0.05 considered statistically significant.
