# Supplementary material for: Genotypic Diversity and Pathogenic Potential of Clinical and Environmental Vibrio parahaemolyticus Isolates From Brazil
Source: Front Microbiol. 2021 Mar 12;12:602653. doi: 10.3389/fmicb.2021.602653 (PMC7994283; doi:10.3389/fmicb.2021.602653)
Supplement: Supplementary file 4 [file Image_4.pdf]

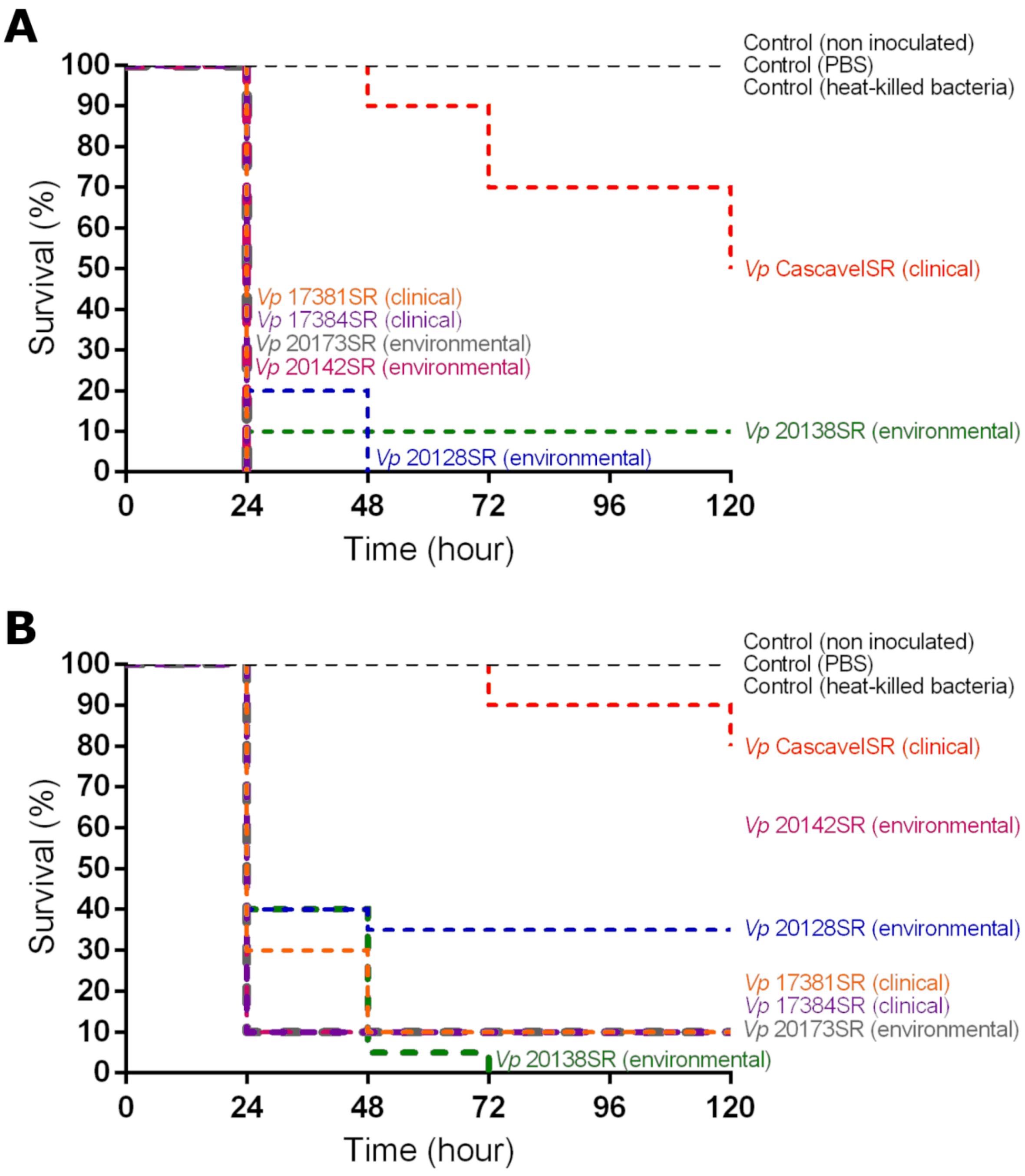

Supplementary Figure S4. Survival curves of *G. mellonella* larvae inoculated with (A) 10^6 CFU/larva and (B) 10^5 CFU/larva of each of the seven *V. parahaemolyticus* strains. Each group contained n = 20 larvae per dose of each strain. Three control groups were used: non inoculated, inoculated with PBS and heat-killed bacteria, n = 10 larvae per group. Survival curves of larvae in each group were generated using the Kaplan-Meier method (Kaplan and Meier, 1958) and compared using the log-rank test (Mantel, 1966; Peto and Peto, 1972), with p-values < 0.05 considered statistically significant.
