# Supplementary material for: Genotypic Diversity and Pathogenic Potential of Clinical and Environmental Vibrio parahaemolyticus Isolates From Brazil
Source: Front Microbiol. 2021 Mar 12;12:602653. doi: 10.3389/fmicb.2021.602653 (PMC7994283; doi:10.3389/fmicb.2021.602653)
Supplement: Supplementary file 5 [file Table_1.DOCX]

**Supplementary Table S1. Pairwise comparisons of the nucleotide (below diagonal) and deduced amino acid sequences (above diagonal) of *vpadF* genes from the *V. parahaemolyticus* isolates analyzed in this work and the reference strains RIMD2210633 e BB22OP**

| ***Vibrio***  **strains** | ***Vp***  **Cascavel** | ***Vp***  **17381** | ***Vp***  **17384** | ***Vp***  **20128** | ***Vp***  **20138** | ***Vp***  **20142** | ***Vp***  **20173** | ***Vp***  **RIMD2210633** | ***Vp***  **BB22OP** |
| --- | --- | --- | --- | --- | --- | --- | --- | --- | --- |
| ***Vp* Cascavel** |  | 98 | 98 | 99 | 98 | 98 | 98 | 98 | 99 |
| ***Vp* 17381** | 99 |  | 100 | 98 | 98 | 99 | 99 | 100 | 99 |
| ***Vp* 17384** | 99 | 100 |  | 98 | 98 | 99 | 99 | 100 | 99 |
| ***Vp* 20128** | 99 | 98 | 98 |  | 98 | 98 | 98 | 98 | 98 |
| ***Vp* 20138** | 98 | 99 | 99 | 98 |  | 98 | 98 | 98 | 99 |
| ***Vp* 20142** | 98 | 99 | 99 | 98 | 99 |  | 100 | 99 | 99 |
| ***Vp* 20173** | 98 | 99 | 99 | 98 | 99 | 100 |  | 99 | 99 |
| ***Vp* RIMD2210633** | 99 | 100 | 100 | 98 | 99 | 99 | 99 |  | 99 |
| ***Vp* BB22OP** | 99 | 99 | 99 | 98 | 99 | 99 | 99 | 99 |  |
